# When Gold Is Not Enough: Platinum Standard of Quantum Chemistry with *N*^7^ Cost

**DOI:** 10.1021/acs.jctc.2c00460

**Published:** 2022-10-31

**Authors:** Michał Lesiuk

**Affiliations:** Faculty of Chemistry, University of Warsaw, Pasteura 1, Warsaw 02-093, Poland

## Abstract

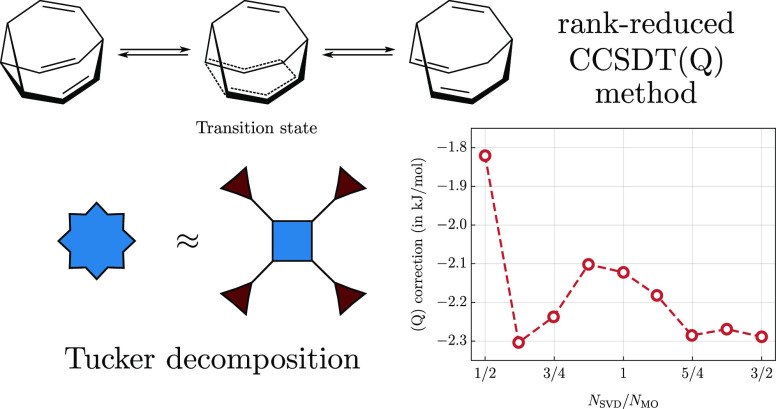

In this paper, we extend the rank-reduced coupled-cluster formalism to the calculation of non-iterative energy corrections due to quadruple excitations. There are two major components of the proposed formalism. The first is an approximate compression of the quadruple excitation amplitudes using the Tucker format. The second is a modified functional used for the evaluation of the corrections which gives exactly the same results for the exact amplitudes, but is less susceptible to errors resulting from the aforementioned compression. We show, both theoretically and numerically, that the computational cost of the proposed method scales as the seventh power of the system size. Using reference results for a set of small molecules, the method is calibrated to deliver relative accuracy of a few percent in energy corrections. To illustrate the potential of the theory, we calculate the isomerization energy of *ortho*/*meta* benzyne (C_6_H_4_) and the barrier height for the Cope rearrangement in bullvalene (C_10_H_10_). The method retains a near-black-box nature of the conventional coupled-cluster formalism and depends on only one additional parameter that controls the accuracy.

## 1 Introduction

Tensor decomposition has long been an active area of research in the field of applied mathematics, with successful applications in many branches of science, see ref (1) for an exhaustive review. In recent years, tensor decomposition techniques have been embraced by the quantum chemistry community, as exemplified by the development of the tensor hypercontraction (THC) format of the electron repulsion integrals.^2−5^ Pioneering applications to electronic structure methods such as MP2, MP3, random-phase approximation, and coupled cluster have also been reported.^6−17^ The primary motivation for applying tensor decompositions to quantum-chemical methods are reductions in terms of computational cost and storage requirements. With a proper calibration, these benefits are attainable with an insignificant accuracy loss and without compromising the black-box nature of the parent theoretical method. However, we would like to point out that from the point of view of quantum chemistry, there is an additional potential application of tensor decomposition techniques which has been largely untapped thus far. It is related to the interpretative power of such techniques, exploiting the fact that they can automatically extract important information about the system even from a complicated wave function Ansätz, with minimal human oversight.^1^

The coupled-cluster (CC) theory^18,19^ is a particularly promising candidate for applying tensor decomposition schemes. In all CC variants, the wave function is parametrized by a set of cluster amplitudes which can be viewed as multi-dimensional tensors with indices referring to the occupied and virtual orbital sets. Storage and manipulation of these tensors constitutes the main bottleneck in CC calculations for large molecular systems. To address this issue, we have recently introduced^14^ an approximate CC theory including single, double, and triple excitations (CCSDT)^20,21^ where the triply excited amplitudes tensor is represented in the Tucker-3 format.^22,23^ The increased flexibility offered by this decomposition enables to reduce the scaling of the approximate method by a factor quadratic in the system size in comparison with the exact CCSDT. At the same, accuracy levels of up to 0.1 kJ/mol are reachable in typical applications to chemical problems.

Despite these developments being promising, one may argue from a pragmatic standpoint that being able to reproduce the CCSDT results accurately, even at a significantly reduced cost, is not sufficient for general-purpose applications in thermochemistry, chemical kinetics, molecular interactions, and so forth. In fact, it is well-documented that in some applications, the CCSDT method does not improve the accuracy (in relation to FCI) over the “gold standard” CCSD(T) to a degree that would justify the drastic increase in the computational costs.^24−29^ The reason for this counterintuitive behavior is an accidental, yet systematic, cancellation of errors observed at the CCSD(T) level of theory for “well-behaved” systems. There are two major components of the post-CCSD(T) contribution: (i) the correction due to the inexact treatment of triple excitations and (ii) the correction due to the missing quadruple excitations. It turns out that these two components are often of opposite signs and hence a degree of cancellation occurs. The lesson learned is that the quadruple excitations play a significant role in the ≈1 kJ/mol accuracy range and must be included alongside the full treatment of triple excitations to provide a balanced description.

The importance of quadruple excitations in accurate theoretical studies was recognized in the literature a long time ago. Unfortunately, their complete inclusion by means of the full CCSDTQ theory^30−33^ is prohibitive for molecules comprising more than a few atoms, assuming a decent-quality basis set is used. This prompted research into more affordable methods that are able to account for the quadruple excitations in an approximate, yet still reliable, way. Several families of such methods were proposed,^34−41^ both iterative and non-iterative, based either on the ordinary Møller–Plesset perturbation theory or various effective Hamiltonian approaches, and employing either CCSD or CCSDT wave functions as the starting point. A more detailed technical discussion of these methods is given in subsequent sections. In this work, we concentrate primarily on the CCSDT(Q) theory introduced by Bomble et al.^37^ which has become the de facto standard in high-accuracy quantum chemical calculations. Due to a good balance between the accuracy and computational costs, it is a member of various composite electronic structure protocols and is implemented in several program packages available for public use. In many applications, the CCSDT(Q) theory is considered to be the “platinum standard” of quantum chemistry^29^—the next rung of the CC ladder above CCSD(T) striking a balance between the accuracy and computational costs. Computation of the (Q) correction is usually 1–2 orders of magnitude less computationally intensive than the complete CCSDTQ calculations. Despite this drastic reduction, the range of applicability of the CCSDT(Q) theory to polyatomic molecules remains limited as a result of steep *N*^9^ scaling of the computational costs with the system size, *N*.

This work is a continuation of a series of papers^11,14,16,17^ where tensor decomposition techniques are applied as a tool to reduce the cost of high-order CC methods. In this part, we introduce a rank-reduced approach to computation of the (Q) correction. There are two main distinguishing features of the proposed scheme. The first is the compression of the quadruply excited amplitudes using the Tucker format which enables to reduce the immense cost of storing and manipulating the *T*_4_ amplitudes. To achieve the necessary transformation from the full rank to rank-reduced representation of the quadruply excited amplitudes, we develop an iterative method based on a higher-order orthogonal iteration (HOOI) procedure.^42,43^ The second feature is the development of a modified functional used to evaluate the (Q) correction. Due to the variational nature of this functional, it is less sensitive to the errors incurred by the rank-reduced treatment of the CC amplitudes. This enables to evaluate the (Q) correction with a mean relative accuracy of a few percent. Taking into account that the (Q) method itself is able to recover, on average, about 90% of the CCSDTQ–CCSDT energy difference,^44^ these errors are acceptable from a practical point of view. Critically, by properly factorizing the working expression of the proposed method and exploiting the rank-reduced format of the CC amplitudes, it is possible to evaluate the (Q) correction with the *N*^7^ cost. Finally, we report calculations of relative energies for larger systems, demonstrating a broad range of applicability and reliability of the proposed theory. In particular, we study the isomerization energy of *ortho*/*meta* benzyne and the Cope rearrangement in a bullvalene molecule.

## 2 Theory

### 2.1 Preliminaries

In this work, we consider closed-shell systems and employ the canonical restricted Hartree–Fock (HF) determinant, denoted |ϕ_0_⟩, as the reference wave function in the CC theory. The HF orbital energies are denoted by ϵ_p_. For brevity, we also introduce the following conventions:  and  for arbitrary operators *A* and *B*. Unless explicitly stated otherwise, the Einstein convention for summation over repeated indices is employed throughout. The standard partitioning of the electronic Hamiltonian, *H* = *F* + *W*, into the sum of the Fock operator (*F*) and the fluctuation potential (*W*) is adopted. The remaining aspects of the notation are summarized in Table 1.

**Table 1:** Details of the Notation Adopted in the Present Work; *O* Is the Number of Active Occupied Orbitals in the Reference and *V* Is the Number of Virtual Orbitals^a^

indices	limit	corresponds to	defining equation
*i*, *j*, *k*, *l*, ···	*O*	active occupied orbitals	
*a*, *b*, *c*, *d*, ···	*V*	unoccupied (virtual) orbitals	
*p*, *q*, *r*, *s*, ···		general orbitals	
*P*, *Q*, ···	*N*_aux_	density-fitting basis set	(*pq*|*rs*) = *B*_*pq*_^*Q*^*B*_*rs*_^*Q*^
*X*, *Y*, *Z*, ···	NSVD	subspace of triply excited amplitudes	*t*_*ijk*_^*abc*^ = *t*_*XYZ*_*U*_*ai*_^*X*^*U*_*bj*_^*Y*^*U*_*ck*_^*Z*^
*A*, *B*, *C*, ···	*N*_qua_	subspace of quadruply excited amplitudes	*t*_*ijkl*_^*abcd*^ = *t*_*ABCD*_*V*_*ai*_^*A*^*V*_*bj*_^*B*^*V*_*ck*_^*C*^*V*_*dl*_^*D*^

a For convenience of the readers, the key defining equations are included in the last column.

The method for evaluation of non-iterative quadruples correction reported in this work builds upon the SVD–CCSDT theory introduced in ref (14). The electronic wave function underlying the SVD–CCSDT method is given by  with *T*_SVD_ = *T*_1_ + *T*_2_ + *T*_3_^SVD^. The *T*_1_ and *T*_2_ operators have the same form as in the usual CCSDT theory
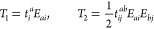
1where *t*_*i*_^*a*^ and *t*_*ij*_^*ab*^ are the cluster amplitudes, and *E*_*pq*_ = *p*_α_^†^*q*_α_ + *p*_β_^†^*q*_β_ are the spin-adapted singlet orbital replacement operators.^45^ The triply excited component of the cluster operator is approximated as
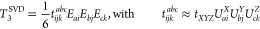
2The quantities *U*_*ai*_^*X*^ are obtained by a procedure described in ref (17) and are fixed during the CC iterations. The remaining unknown quantities (*t*_*i*_^*a*^, *t*_*ij*_^*ab*^, and *t*_*XYZ*_) are found by projecting  onto a proper subset of excited determinants and solving the resulting non-linear equations. The dimension of the compressed amplitudes tensor *t*_*XYZ*_ is denoted by *N*_SVD_, see Table 1, and it scales linearly with the system size. Note that this tensor is supersymmetric, that is, invariant to any permutation of the indices *X*, *Y*, and *Z*.

As a computationally convenient representation of the electron repulsion integrals, we employ the density-fitting approximation^46−50^
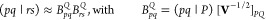
3where (*pq*|*P*) and *V*_*PQ*_ = (*P*|*Q*) are the three-center and two-center electron repulsion integrals, respectively. Because the Coulomb metric is used in eq 3 for the determination of density-fitting coefficients, this formula is automatically “robust” in the sense that the error in the integrals is quadratic in the density errors.^48^ The capital letters *P* and *Q* are employed in the present work for the elements of the auxiliary basis set. The number of auxiliary basis set functions is denoted by the symbol *N*_aux_. By construction, *N*_aux_ scales linearly with the size of the system. In all calculations reported in this work, the error in relative energies caused by the density-fitting approximation was negligible in comparison with other uncertainties. This is consistent with other studies on this topic found in the literature.^51,52^

### 2.2 Non-Iterative Quadruple Corrections

The problem of economical inclusion of quadruple excitation effects in the CC theory was first considered by Kucharski, Bartlett, and collaborators.^34−36,53−56^ They introduced a non-iterative method, denoted CCSDT[Q] or simply [Q] in the present work, based on the standard Møller–Plesset perturbation theory where the Hartree–Fock determinant serves the role of the zeroth-order wave function. The quadruple excitation cluster operator *T*_4_ is obtained from an approximate formula
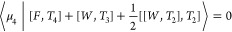
4where μ_4_ stands for an appropriate string of quadruple excitation operators, that is, μ_4_ = *E*_*ai*_*E*_*bj*_*E*_*ck*_*E*_*dl*_, and hence ⟨μ_4_| denotes projection onto the quadruply excited configurations. A similar notation is used below also for lower-order excitations, for example, μ_3_ = *E*_*ai*_*E*_*bj*_*E*_*ck*_. As the Fock operator is diagonal in the canonical orbital basis, eq 4 can be explicitly solved to get the quadruply excited amplitudes in a closed-form
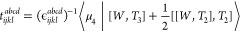
5where ϵ_*ijkl*_^*abcd*^ = ϵ_*i*_ + ϵ_*j*_ + ϵ_*k*_ + ϵ_*l*_ – ϵ_*a*_ – ϵ_*b*_ – ϵ_*c*_ – ϵ_*d*_ is the four-particle energy denominator. The CCSDT[Q] correction to the energy (abbreviated as *E*_[Q]_) originating from the missing quadruple excitations then reads
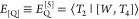
6where the superscript [5] indicates that the term enters in the fifth order of the Møller–Plesset perturbation theory. An alternative method to account for the quadruple excitations was presented by Bomble et al.^37^ who employed Löwdin’s partitioning of the CC EOM Hamiltonian.^57^ This method is nowadays most commonly referred to as CCSDT(Q). The main difference between this approach and the pioneering developments of Kucharski and Bartlett is that the CCSDT wave function, rather than the Hartree–Fock determinant, is employed as the zeroth-order wave function. The resulting energy correction, denoted by *E*_(Q)_, uses the same formula 5 for the quadruply excited amplitudes, but is given by the sum of two terms
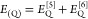
7The former term is the same as in the CCSDT[Q] method, eq 6, while the latter reads
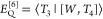
8As suggested by the notation, the term *E*_Q_^[6]^ is of the sixth order in the usual perturbation theory and hence it was neglected in the CCSDT[Q] method. However, it has been shown^37^ that the importance of the *E*_Q_^[6]^ contribution is much larger than its formal order would suggest. Only in the basis sets of double-zeta quality, the term *E*_Q_^[5]^ is dominating and the contribution from *E*_Q_^[6]^ is typically smaller by an order of magnitude. This changes when the size of the basis set is increased to triple-zeta or larger. The terms *E*_Q_^[5]^ and *E*_Q_^[6]^ are then of a similar magnitude, with the latter even becoming dominant in some cases. As an immediate consequence, the relative accuracy of the CCSDT[Q] method (in comparison to CCSDTQ) deteriorates with the increase in basis set size. The accuracy level of the (Q) correction, on the other hand, was found to be remarkably consistent at least up to quintuple-zeta basis sets.^37^ In the present work, we concentrate primarily on the implementation of the CCSDT(Q) method as a way of incorporating the effects of quadruple excitations into the rank-reduced CC formalism. To further justify this choice, below we provide a short survey of other available methods. We concentrate on closed-shell systems and hence open-shell generalizations are not discussed.

In the factorizable [Q] method,^35^ denoted accordingly by [Q_f_], one employs the factorization theorem^58^ to get rid of the four-particle denominator in evaluation of the *E*_Q_^[5]^ term. While this factorization is only approximate in the case of CCSDT amplitudes entering eq 6, the quality of this approximation is usually excellent. The main advantage of the [Q_f_] method is the reduced scaling of the computational cost with the system size. While the exact computation of the [Q] correction scales as *N*^9^, evaluation of the factorizable variant [Q_f_] can be accomplished with the *N*^7^ cost. Unfortunately, the [Q_f_] method itself is an approximation to the [Q] correction, and hence, it is bound to suffer from the same basis set dependency problems. To the best of our knowledge, analogous factorization cannot be accomplished for the *E*_Q_^[6]^ term that involves projection onto the triply excited amplitudes.

The second family of non-iterative quadruples corrections retains different parametrizations of the left- and right-hand-side CC wave functions.^38^ The resulting CCSDT[Q]_Λ_ and CCSDT(Q)_Λ_ methods offer a noticeable improvement in terms of the accuracy in comparison to their conventional counterparts described above. However, this comes at a cost of evaluating the so-called CC Lagrangian which is not available at present for the rank-reduced CCSDT method and requires a separate study. Next, we discuss the recently introduced CCSDT(Q-*n*) family of methods derived from Lagrangian-based perturbation theory, treating CCSDT as the zeroth-order wave function.^40,41^ This framework is free from size-inconsistency problems encountered in the preceding EOM-like approaches and has been shown to converge rapidly to the exact CCSDTQ limit. While CCSDT(Q-2) is not competitive with the CCSDT(Q) theory, the improved CCSDT(Q-3) variant offers an excellent accuracy level.^44^ Unfortunately, the computational cost of the CCSDT(Q-3) method is comparable to a single CCSDTQ iteration (*N*^10^ scaling) and hence it is beyond the scope of the present work. Last but not least, the renormalized and completely renormalized approaches developed by Piecuch and collaborators^59−62^ are derived using the so-called CC method of moments. They drastically improve the accuracy for systems with significant multireference character, but for systems dominated by a single reference determinant, the results are similar.

### 2.3 Quadratic (Q) Functional: Exact Formulation

In the rank-reduced context, the formulation of the (Q) correction based on eqs 6–8 has a significant disadvantage. It stems from the fact that these equations were derived assuming that the *T*_1_, *T*_2_, and *T*_3_ amplitudes come from the exact CCSDT theory and *T*_4_ amplitudes are obtained by solving eq 4 without further approximations. In the rank-reduced formalism, these assumptions do not hold; for example, the *T*_3_ amplitudes are subject to the Tucker-3 compression, see eq 2. Unfortunately, if approximate cluster amplitudes are used to evaluate eqs 6 and 8, the error in the (Q) correction is roughly proportional to the error of the amplitudes. In other words, there exists an approximate linear relationship connecting the average error in the amplitudes and the error in the (Q) correction. To avoid this problem and to guarantee that the latter error vanishes more rapidly as the accuracy of the amplitudes is improved, we propose a different functional for the evaluation of the (Q) correction. The general form of the new functional, denoted  further in the text, reads
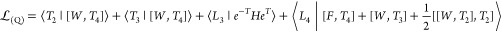
9where *L*_3_ and *L*_4_ are two new auxiliary operators which assume the standard form
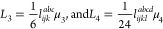
10and the new amplitudes *l*_*ijk*_^*abc*^ and *l*_*ijkl*_^*abcd*^ are yet to be determined. The proposed functional has to fulfill two main theoretical requirements in order to be useful in the rank-reduced context:

- If the exact CCSDT amplitudes together with *T*_4_ amplitudes calculated from eq 4 are used, the new functional gives strictly identical results as the original formulation based on eqs 6 and 8;
- The error of the (Q) correction evaluated using the new functional is quadratic in the error of the *T*_3_/*L*_3_ and *T*_4_/*L*_4_ amplitudes.

The motivation behind the first requirement is to enforce that in the limit of the complete triple excitation subspace in eq 2, that is, when the SVD–CCSDT method is equivalent to the conventional CCSDT, the exact (Q) correction is recovered. Regarding the second requirement, the goal is to reduce the impact of the approximations adopted in the treatment of *T*_3_ and *T*_4_ amplitudes on the accuracy of the (Q) correction. However, one might ask why the quadratic error property is enforced only with respect to the *T*_3_ and *T*_4_ amplitudes, disregarding the *T*_2_ amplitudes that enter eq 6 directly and eq 8 indirectly via the *T*_4_ operator. The justification is purely pragmatic and is based on a numerical observation that the *T*_2_ amplitudes obtained from the SVD–CCSDT method are sufficiently accurate for the purposes of evaluating eqs 6 and 8. In fact, we verified that even the use of CCSD *T*_2_ amplitudes results in acceptable errors. This finding is not entirely surprising as similar arguments are used in the derivation of the aforementioned factorizable approximation to the *E*_Q_^[5]^ term. All in all, while the second requirement given above can be strengthened to include the *T*_2_ amplitudes as well, we found no practical reason to justify such choice, taking into account the increased complexity of the resulting formalism.

It is straightforward to verify that for any *L*_3_ and *L*_4_ operators, the  functional automatically fulfills the first requirement given in the previous paragraph. In fact, when the exact CCSDT amplitudes are inserted into the above formula, the third term vanishes identically as a consequence of the CCSDT stationary condition for the triple excitation amplitudes, ⟨μ_3_|*e*^–*T*^*He*^*T*^⟩ = 0. Similarly, if the *T*_4_ amplitudes are determined from eq 4 without approximations, the fourth term included in  also vanishes, as it is a projection of eq 4 onto some set of *L*_4_ amplitudes.

In order to satisfy the second requirement discussed above, we demand that the  functional is stationary with respect to variations in the *T*_3_, *T*_4_, *L*_3_, and *L*_4_ amplitudes. In other words, we impose a condition that the first derivative of eq 9 with respect to each of these amplitudes separately is zero. Differentiation with respect to the *L*_3_ and *L*_4_ amplitudes returns back the stationary conditions and eq 4, respectively, and hence this brings no new information into the formalism. By differentiating  with respect to the *T*_3_ and *T*_4_ amplitudes, respectively, and setting the resulting equations to zero, one obtains
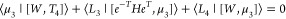
11and
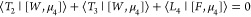
12respectively. The latter equation can be directly solved to obtain the *L*_4_ amplitudes. Upon inserting the results into eq 11, it becomes a system of linear equations with the *L*_3_ amplitudes being the only unknowns. Therefore, eqs 11 and 12 completely determine the auxiliary *L*_3_ and *L*_4_ operators and hence enable calculation of the modified (Q) functional, eq 9.

With eqs 11 and 12 at hand, it remains to show that the quantity  indeed fulfills the second condition discussed above. The simplest way to achieve this is to employ the chain rule of differentiation and exploit the stationary conditions (eq 9). However, in the Supporting Information, we provide a more detailed derivation that has the advantage of providing a rigorous error estimation, namely
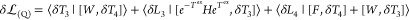
13where *T*^ex^ denotes the exact CCSDT amplitudes, while δ*T*_3_ is an error in the *T*_3_ operator (analogous notation is used for the remaining quantities). It is straightforward to verify that each term of the above formula is quadratic in the combined powers of δ*T*_3_, δ*L*_3_, δ*T*_4_, and δ*L*_4_. This proves that the proposed functional  satisfies the second requirement introduced at the beginning of this section.

### 2.4 Quadratic (Q) Functional: Approximations

In order to make calculations based on the quadratic (Q) functional feasible, approximations need to be introduced to the exact formalism presented in the previous section. However, we stress that in order to retain the desirable properties of the  functional, no approximations are made to its formal definition given by eq 9. Instead, we adopt several simplifications to the equations that determine the *T*_3_, *T*_4_, *L*_4_, and *L*_3_ operators, as described in detail in this section. Due to the quadratic nature of the  functional, these approximations are expected to have a small impact on the accuracy of the (Q) correction.

Starting with the *T*_3_ operator, it is given by the approximate form (eq 2), inherited after the SVD–CCSDT theory, with the amplitudes compressed using the Tucker-3 format. We adopt no further approximations to this quantity.

Moving to the *T*_4_ operator, the handling and storage of the full-rank quadruply excited amplitudes given by eq 5 constitutes the major bottleneck of the exact formalism. To overcome this obstacle, we approximately cast the quadruply excited amplitudes (eq 5) in a rank-reduced form employing the Tucker format
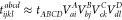
14which is fully analogous to the rank-reduced form of the triply excited amplitudes, cf. eq 2. As the expansion basis vectors *V*_*ai*_^*A*^ are distinct from their counterparts used in eq 2, we employ the capital letters *A*, *B*, *C*, ··· to denote the quantities that relate to the quadruply excited amplitudes. The dimension of the core tensor *t*_*ABCD*_ in eq 14 is referred to as *N*_qua_. By analogy with the findings for the doubly and triply excited amplitudes represented in the Tucker-*n* format, we assume that *N*_qua_ scales linearly with the system size. A numerical demonstration of this condition is presented in Section 3.2. The conversion of the full-rank quadruply excited amplitudes *t*_*ijkl*_^*abcd*^ into the compressed form (eq 14) is non-trivial. To accomplish this task, we propose a novel algorithm based on HOOI. Details of this procedure are described in the next section, along with the analysis of the computational costs and scaling with the system size.

Next, we consider the auxiliary operator *L*_4_, which is defined by eq 12. To facilitate efficient evaluation of this quantity, we adopt two levels of approximations. First, in eq 12, we neglect the term that involves the triply excited amplitudes, namely, . The justification of this approximation is rooted in the standard Møller–Plesset perturbation theory, where the *T*_2_ operator enters in the first-order perturbed wave function, while the *T*_3_ operator appears in the second order. By the same token, we expect the contribution of the  to be dominating, while the neglected term constitutes a relatively minor correction. The neglect of the term  leads to the modified expression
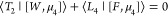
15which can easily be solved directly by exploiting the diagonal nature of the canonical Fock operator, giving
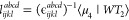
16In the Supporting Information, we provide an explicit formula for this quantity expressed through the basic CC amplitudes and two-electron integrals. The second level of approximation adopted for *L*_4_ is the same as for the *T*_4_, that is, rank-reduction to the Tucker-4 format
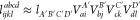
17where the primes have been added to underline that the expansion basis is different from that of eq 14. Similarly as for the *T*_4_ operator, in the next section, we provide technical details of the HOOI procedure used to determine the rank-reduced *L*_4_ amplitudes.

Finally, let us consider the *L*_3_ operator for which the approximation scheme is somewhat more involved and consists of two steps. In the first step, we neglect the fluctuation potential *W* in the similarity-transformed Hamiltonian present in eq 11 and set *e*^–*T*^*He*^*T*^ ≈ *e*^–*T*^*Fe*^*T*^. This leads to the modified formula
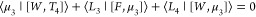
18where we have additionally exploited the fact that , which is straightforward to prove using the BCH expansion. By exploiting the properties of the Fock operator, explicit solution of this equation is written as
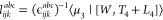
19where the equality  has been used for convenience sake.

To introduce the second layer of approximation, we note that the third term in eq 18 is dominating in comparison with the first term. This is again justified by perturbation theory arguments; by comparing eqs 15 and 4, we see that *L*_4_ is a second-order quantity, while *T*_4_ is a third-order quantity. Therefore, it is tempting to neglect the  term altogether in eq 18, in a similar spirit as in the previous paragraph where approximations of the *L*_4_ operator were discussed. We considered this approach in the preliminary stage of the implementation and verified that it indeed delivers a decent accuracy level. However, there exists an alternative approach to approximating the *L*_3_ operator which is based on the Tucker format
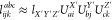
20where the primes indicate that the quantities  are distinct from the expansion basis used for the triply excited amplitudes in eq 2. Similarly as for other quantities, HOOI is used to bring *l*_*ijk*_^*abc*^ into the decomposed form (eq 20). However, as a cost-saving measure, we introduce a simplification: the expansion basis  is found by decomposing an approximate form of the *l*_*ijk*_^*abc*^, namely
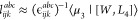
21where the term including the *T*_4_ operator has been neglected, cf. eq 19. Once the quantities  are found, the core tensor  is found by projection
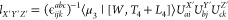
22Note that in this step, the full form of the *L*_3_ operator is used, without any approximations in comparison with eq 19. We found that this hybrid approach reduces the computational cost of decomposing the amplitudes *l*_*ijk*_^*abc*^ considerably without affecting the accuracy of the decomposition (eq 20). This can be explained by noting that the term involving the *T*_4_ is numerically minor. Therefore, the basis found using the decomposition of the approximate formula, eq 20, is able to accommodate both terms accurately, despite the *T*_4_ being absent in the optimization procedure.

To study the impact of the proposed approximations, we carried out calculations for small molecular systems from Section 3.3. In the Supporting Information, we provide detailed results for the most representative case using the cc-pVDZ, cc-pVTZ, and cc-pVQZ basis sets. These data show that the neglected terms are numerically small and some additional approximations proposed above have a small impact on the accuracy of the proposed formalism.

Finally, let us point out that, in general, the dimensions of the quadruple excitation subspace (*N*_qua_) used for compression of the *t*_*ijkl*_^*abcd*^ and *l*_*ijkl*_^*abcd*^ amplitudes, that is, in eqs 14 and 17, can be different. Similarly, different sizes of the triple excitation subspace (*N*_SVD_) may be used for *T*_3_ and *L*_3_ operators. However, in a set of preliminary calculations, we found that near-optimal results are attained when the same size of the quadruple excitation subspace is used for *T*_4_ and *L*_4_, and the same size of triple excitation subspace for *T*_3_ and *L*_3_. Therefore, we use a single parameter *N*_qua_ to denote the length of the expansion in both eqs 14 and 17, and similarly *N*_SVD_ for both eqs 20 and 2. Gains in terms of accuracy achieved by lifting these restrictions do not justify the corresponding increase of the technical complexity of the formalism.

### 2.5 Compression of the Excitation Amplitudes

As mentioned in the previous section, the decomposition of the triply and quadruply excited amplitudes, required to evaluate the approximate form of the quadratic functional detailed in Section 2.4, is achieved using the HOOI procedure. In this section, we provide details of this procedure, both theoretical and technical. We concentrate primarily on the decomposition of the *t*_*ijkl*_^*abcd*^ tensor as this is the most problematic quantity. However, extension of this procedure to the amplitudes present in the *L*_3_ and *L*_4_ operators is briefly discussed at the end of the present section, and further technical details are presented in the Supporting Information.

In the HOOI procedure, the decomposition of the amplitudes is achieved by minimization of the following cost function
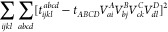
23subject to the condition that the *V*_*ai*_^*A*^ vectors are column orthonormal, that is
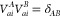
24The constraint (eq 24) is imposed without the loss of generality, because any linear transformation among the *V*_*ai*_^*A*^ vectors can be counteracted by changing the values of the core tensor, leaving the cost function unaffected. For a fixed expansion length (*N*_qua_) the least-squares problem (eq 23) is solved by HOOI which in the present case proceeds as follows. Assuming that an approximate solution *V*_*ai*_^*A*^ of the minimization problem (eq 23) is known, one forms an intermediate quantity
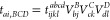
25which is a partial projection of the full-rank tensor onto the current subspace. Next, the truncated singular value decomposition (SVD) of the *t*_*ai*,*BCD*_ tensor is computed. The left-singular vectors corresponding to *N*_qua_ the largest singular values form the updated expansion vectors, *V*_*ai*_^*A*^. Note that by the virtues of the SVD procedure, the new vectors automatically obey the orthonormality constraint (eq 24). This basic iteration process is repeated until the convergence criterion is met; a convenient choice of the stopping criteria is discussed further in the text. Note that during the HOOI procedure, the core tensor *t*_*ABCD*_ does not have to be formed explicitly. Nonetheless, it is obtained straightforwardly as
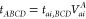
26owing to the orthonormality of the expansion vectors, *V*_*ai*_^*A*^.

The basic HOOI procedure described above constitutes a serviceable method. Unfortunately, due to large dimensions of the *t*_*ai*,*BCD*_ matrix, namely, *OV* × *N*_qua_^3^, the SVD step of this algorithm is too expensive for large-scale applications. Therefore, we introduce a modification of the HOOI procedure where instead of the *t*_*ai*,*BCD*_ matrix, the following quantity is computed
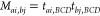
27Let us recall that for any rectangular matrix **M**, its left singular-vectors coincide with the eigenvectors of the normal matrix **MM**^T^. Therefore, the updated expansion vectors *V*_*ai*_^*A*^ can equivalently be obtained by diagonalizing the *M*_*ai*,*bj*_ matrix and retaining the eigenvectors corresponding to the largest eigenvalues (which are non-negative by construction). The dimensions of the *M*_*ai*,*bj*_ matrix are *OV* × *OV* and hence it admits eigen decomposition in *N*^6^ time, a significant improvement over the SVD of the *t*_*ai*,*BCD*_ matrix. As a by-product, the modification of the HOOI procedure described above solves the memory bottleneck related to the storage of the complete *t*_*ai*,*BCD*_ matrix (*N*^5^ memory chunk). In our implementation, the *t*_*ai*,*BCD*_ matrix is calculated in batches with one of the *B*, *C*, *D* indices fixed. The batches are then immediately used to compute the contribution to *M*_*ai*,*bj*_ without accumulation of the full *t*_*ai*,*BCD*_ matrix. The storage requirements are reduced in this way to the level of *OVN*_qua_^2^ ∝ *N*^4^.

It remains to discuss two technical aspects of the HOOI algorithm, that is, the choice of the stopping criteria and the starting values. As discussed at length in ref (17), an appropriate stopping condition is obtained by monitoring the norm of the core tensor
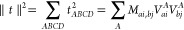
28where the second equality follows from the orthonormality of the *V*_*ai*_^*A*^ vectors. The HOOI procedure is ended when the relative difference in ∥*t*∥ between two consecutive iterations falls below a predefined threshold, ϵ. The threshold value of ϵ = 10^–6^ is sufficient in most applications and has been adopted in the present work. The cost of computing ∥*t*∥ is negligible in comparison with other parts of the algorithm. The reason why this straightforward procedure performs well in practice is a consequence of the fact that the HOOI algorithm can be reformulated as a maximization of the norm of the core tensor instead of minimization of eq 23, see refs (42) and (63).

The problem of the starting values is solved simply by setting *V*_*ai*_^*A*^ as equal to *U*_*ai*_^*X*^ that correspond to the largest absolute diagonal values of *t*_*XYZ*_, see eq 2. We found this procedure to be entirely satisfactory in practice and convergence of the HOOI procedure is achieved typically within only 5–10 iterations to accuracy of ϵ = 10^–6^. The sole exception from this rule occurs in calculations with extremely small *N*_qua_, but it is questionable whether they are of any practical importance. The major steps of the HOOI algorithm are summarized in Algorithm 1.
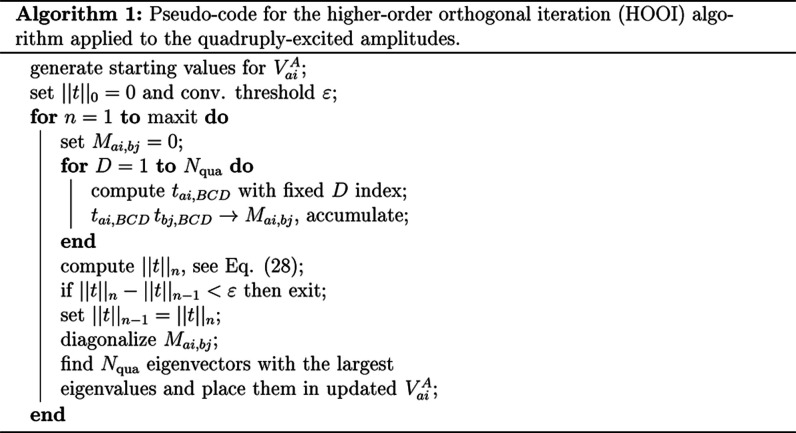


According to the above discussion, the critical part of the HOOI algorithm is the calculation of partly projected quadruply excited amplitudes, eq 25. Without invoking any approximations, the computational cost of this step is proportional to *N*^9^, even under the assumption that *N*_qua_ scales linearly with the system size. This offers no practical advantages over the available conventional algorithms for calculation of the quadruples corrections. The main reason behind this steep scaling is the presence of the four-particle energy denominator in eq 5 which has to be eliminated in order to enable any scaling reduction. To this end, we employ the discrete Laplace transformation (LT) technique
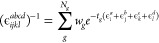
29where *t*_*g*_ and *w*_*g*_ are the quadrature nodes and weights, respectively, *N*_*g*_ is the size of the quadrature, and ϵ_*i*_^*a*^ = ϵ_*i*_ – ϵ_*a*_. For the two-particle energy denominator, this method was first proposed by Almlöf^64^ in the context of the MP2 theory, but since then, it has been successfully used in combination with other electronic structure methods.^65−70^ In this work, we employ the min–max quadrature proposed by Takatsuka and collaborators^71−73^ for the choice of *t*_*g*_ and *w*_*g*_. The number of quadrature points in eq 29 is independent of the system size, that is, *N*_*g*_ ∝ *N*^0^. To the best of our knowledge, this is the first application of the LT technique to the four-particle energy denominator in the CC theory.

The quadruply excited amplitudes defined by eq 5 are given by the following explicit expression
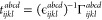
30where
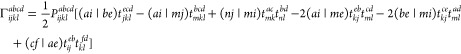
31The permutation operator *P*_*ijkl*_^*abcd*^ in the above formula reads
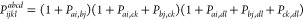
32and *P*_*ai*,*bj*_ denotes the basic transposition operator that exchanges pairs of indices *i* ↔ *j* and *a* ↔ *b* simultaneously. By employing the LT technique, we rewrite eq 25 in the form
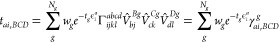
33with  and . Within this formulation of the problem, the overall scaling of assembling the quantity *t*_*ai*,*BCD*_ can be reduced to the level of *N*^6^. To illustrate this, let us consider a term (*bj*|*ae*)*t*_*ikl*_^*ecd*^ obtained by the permutation of indices from the first term in eq 31. In the conventional implementation, this term scales as *O*^4^·*V*^5^. However, a contribution of this term to the quantity γ_*ai*,*BCD*_^*g*^ required in eq 33 can be factorized as
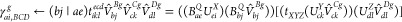
34where we have exploited the density-fitting factorization of the electron repulsion integrals, see eq 3, and Tucker factorization of the triply excited amplitudes tensor given by eq 2. The parentheses included in the above formula indicate the order of operations and should be read starting from the innermost bracket. By following the optimal order of contractions, one can show that the most costly step scales as *N*_*g*_*OVN*_SVD_*N*_qua_^3^ ∝ *N*^6^. Similar factorizations are possible for the remaining terms appearing in eq 31 and in every case the scaling is proportional to *N*^6^, albeit with different prefactors. However, the number of terms in eq 31 that have to be factorized is large—144 in total if all permutations resulting from the action of the *P*_*ijkl*_^*abcd*^ are taken into account. Therefore, explicit factorized formulas for the quantity γ_*ai*,*BCD*_^*g*^ are given in the Supporting Information, along with a detailed discussion of possible simplifications.

Let us point out that the cost of the complete HOOI procedure is still asymptotically proportional to *N*^7^ due to the need to assemble the *M*_*ai*,*bj*_ matrix, see eq 27. However, this step can be formulated as a single dgemm matrix–matrix multiplication and hence possesses a relatively small prefactor. Therefore, the calculation of the quantity γ_*ai*,*BCD*_^*g*^, in spite of the *N*^6^ scaling, constitutes the majority of the total cost of the HOOI procedure for systems that can currently be studied.

The presentation given in this section has been focused on the *t*_*ijkl*_^*abcd*^ amplitudes. However, Algorithm 1 is straightforwardly adapted for an analogous decomposition of the remaining quantities, namely, the *l*_*ijk*_^*abc*^ and *l*_*ijkl*_^*abcd*^ amplitudes that parametrize the *L*_3_ and *L*_4_ operators. This is particularly seamless in the latter case as the only change required is the modification of eq 25 without affecting any other steps of Algorithm 1. Efficient evaluation of the modified expression is discussed in the Supporting Information and it is shown that the scaling of this step is *N*^5^, that is, lower than in the case of the *t*_*ijkl*_^*abcd*^ amplitudes. Somewhat more advanced modifications of the HOOI procedure are required for the *l*_*ijk*_^*abc*^ amplitudes. First, instead of eq 25, one calculates , and the matrix *M*_*ai*,*bj*_ required in Algorithm 1 is obtained as *M*_*ai*,*bj*_ = *l*_*ai*,*Y*_^′^_*Z*_^′^*l*_*bj*,*Y*_^′^_*Z*_^′^, cf. eq 27. The remaining steps of the HOOI procedure are not affected. In the Supporting Information, we provide a factorized expression that enables to compute the intermediate quantity *l*_*ai*,*Y*_^′^_*Z*_^′^ with *N*^6^ cost, meaning that the decomposition of the *l*_*ijk*_^*abc*^ amplitudes scales as *N*^6^ overall.

To sum up the theoretical section of the paper, we point out that computation of the  functional involves several steps, some of which have to be performed in a predefined order. For completeness, these steps are summarized in the Supporting Information, identifying the relevant key equations and presenting additional technical details. Importantly, by analyzing the working expressions, we show that all components of the  functional can be evaluated with a cost proportional to *N*^7^ or less. A numerical confirmation of this finding is provided in the next section.

## 3 Numerical Results and Discussion

### 3.1 Computational Details

Unless explicitly stated otherwise, in all calculations reported in this work, the correlation-consistent cc-pV*X*Z basis set from ref (74)is employed. The corresponding density-fitting auxiliary basis sets cc-pV*X*Z-RIFIT were taken from the work of Weigend et al.^75^ In some calculations, specified further in the text, a larger cc-pV5Z-RIFIT basis from ref (76) was employed to minimize the density-fitting error. Pure spherical representation (5d, 7f, etc.) of all Gaussian basis sets is adopted throughout. Density-fitting approximation is used in all correlated calculations unless written otherwise. However, the Hartree–Fock equations are solved using the exact two-electron integrals and hence the canonical HF orbitals are exact within the given one-electron basis. The frozen-core approximation is invoked in all computations reported in this work, unless explicitly stated otherwise. The 1s^2^ core orbitals of the first-row atoms (Li–Ne) are not correlated.

The reference CCSDT(Q) calculations and calculation of zero-point vibrational energies and harmonic frequencies were performed with the CFOUR program package.^77,78^ Some of the more demanding CCSD(T) calculations reported in this work, indicated below, were performed using NWChem program,^79^ version 6.8. The calculations performed using the CFOUR and NWChem programs do not use the density-fitting approximations. All theoretical methods described in this work were implemented in a computer program written specifically for this purpose which is available from the author upon request. The TBLIS library^80^ is used in the code for performing efficient tensor operations. It is worth mentioning that TBLIS natively supports shared-memory multiprocessing (using OpenMP application programming interface in our case) and hence most of the calculations reported in this work are performed in parallel, unless specified otherwise. Speed-ups by approximately a factor of 10 were observed in large-scale calculations on 12 (internode) threads. Beyond this point, overheads related to, for example load balancing and synchronization, become significant and further increase of the number of computing threads leads to diminishing returns. A higher level of parallelization is possible using the MPI standard. This requires to divide the workload into an independent task and, in the present context, it is natural to distribute the *B*_*pq*_^*Q*^ three-center integrals by splitting the index *Q* among the computing nodes. However, this possibility has not been exploited in the present work. Lastly, the current implementation of the proposed theory does not utilize spatial symmetry of the molecules. Therefore, all calculations reported in this work are performed within the C_1_ symmetry group.

To avoid confusion, we briefly touch upon the naming conventions used in this section. The abbreviation SVD–CCSDT designates the iterative rank-reduced CC method introduced in ref (14), and described briefly in Section 2.1, which is based on the Tucker compression of the triple excitation amplitudes, eq 2. The abbreviation SVD–CCSDT+ refers to a method introduced in ref (16). It consists of adding a non-iterative correction that accounts for triple excitations outside the subspace used in SVD–CCSDT calculations. In this way, the error with respect to the exact CCSDT method is reduced, even if the subspace of triple excitations used in eq 2 is small. Note that both SVD–CCSDT and SVD–CCSDT+ methods become functionally equivalent to the exact CCSDT for a sufficiently large value of the *N*_SVD_ parameter that controls the expansion length in eq 2.

### 3.2 Scaling Demonstration

The efficiency of the proposed method hinges on the assumption that the parameter *N*_qua_, which determines the size of the quadruple excitation subspace in eq 14, scales linearly with the system size, *N*. In other words, to retain a constant relative accuracy in the correlation energy as the system size grows, it is sufficient to set *N*_qua_ proportional to *N*. This conjecture is non-trivial because in the limit of the complete quadruple excitation subspace *N*_qua_ scales quadratically with the system size (more precisely, it is equal to the number of occupied times the number of virtual orbitals in the system).

In previous works, the property that dimension of the excitation subspace scales linearly with the system size has been demonstrated numerically for lower-dimensional (two- and three-) analogues of eq 14 using realistic model systems such as linear alkanes or water clusters for which reference CCSD/CCSDT results are available.^12,14,17^ Unfortunately, evaluation of the (Q) correction for systems comprising more than 4–5 non-hydrogen atoms is computationally costly, and hence reference results are not available for these model systems of sufficient size to reach definite conclusions about the behavior of *N*_qua_. As a compromise, we adopt linear hydrogen chains as model systems for the purposes of the scaling demonstration. The chain is composed of H_2_ molecules (bond length 1.4 a.u.) with all hydrogen atoms placed co-linearly, hence the general formula . The distance between centers of mass of two neighboring hydrogen molecules is equal 4.2 a.u. This system behaves as an insulator even in the limit of an infinite chain length, that is, *n* → ∞, which makes the single-reference CC approach valid.

**Figure 1:**
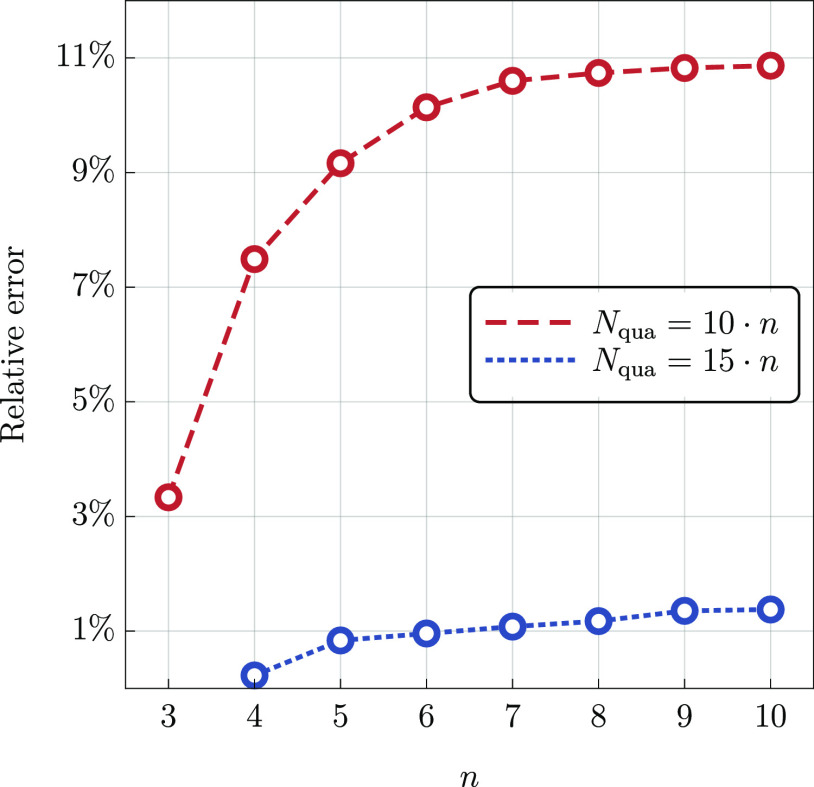
Percentage error in the (Q) correction obtained using the  functional (cc-pVDZ basis set) for the  system, *n* = 3, ···, 10, with *N*_qua_ = 10·*n* and *N*_qua_ = 15·*n*. The exact CCSDT(Q) results are used as a reference.

We performed calculations of the quadruples corrections using the exact CCSDT(Q) method as implemented in CFOUR and the  functional for the  system, *n* = 1, 2, ···, 10, within the cc-pVDZ basis set. We focus solely on the scaling of the *N*_qua_ parameter and hence employ the full space of triple excitations in the SVD–CCSDT calculations preceding the evaluation of the  functional. In Figure 1, we show relative errors in the calculated (Q) correction for a representative value of the quadruple excitation subspace size equal to *N*_qua_ = 10·*n* and *N*_qua_ = 15·*n*, where *n* is the chain length. As the value of *n* increases, the relative error quickly reaches the asymptotic values of approximately 11 and 1%, respectively. In the region beyond *n* ≈ 7, the error is essentially constant with only minor fluctuations of the order of 0.1% or less. This confirms that in order to maintain a constant relative accuracy in the (Q) correction, it is sufficient to make *N*_qua_ proportional to the system size.

Another numerical demonstration necessary to confirm the theoretical findings of the present work is related to the computational cost of evaluating the  functional. The analysis of the working equations of the proposed method given in Sections 2.2–2.4 (and in the Supporting Information) led to the conclusion that all terms in the  functional can be computed with the cost proportional to *N*^7^ in the rate-determining steps. Regarding the decomposition of the *T*_4_ and *L*_4_ amplitudes, they formally share the same *N*^7^ asymptotic cost, but involve only one *N*^7^ step that has a small prefactor. Therefore, we expect that this component of the method would possess, in practice, a cost proportional to *N*^6^. Finally, the decomposition of the *L*_3_ amplitudes was found to scale as *N*^6^.

**Figure 2:**
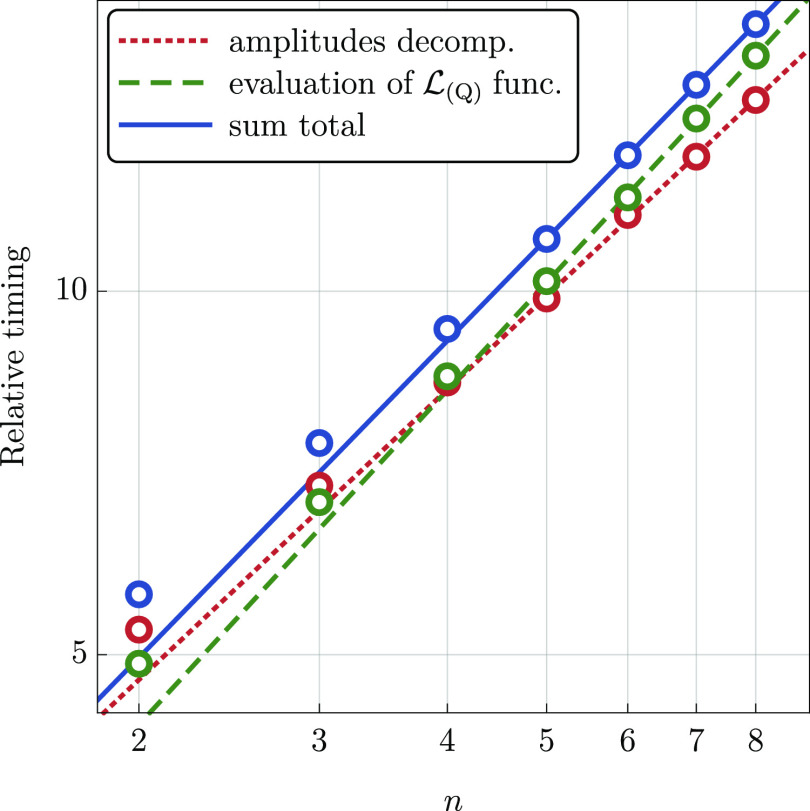
Relative timings of two major computational steps of the proposed formalism (cc-pVDZ basis set) for linear alkanes (C_*n*_H_2*n*+2_) as a function of the chain length, *n*. Logarithmic scale is used on both axes.

To confirm the aforementioned findings numerically, we perform calculations for model systems that can be systematically increased in size. In contrast with the calculations considered at the beginning of this section, the reference CCSDT(Q) calculations are not involved here and hence it is feasible to study a more chemically appealing model system of linear alkanes, C_*n*_H_2*n*+2_. Molecular geometries were taken from ref (14). As an illustrative example, we set *N*_SVD_ = *N*_MO_ and *N*_qua_ = *N*_MO_, where *N*_MO_ is the number of orbitals in the system, so that both parameters increase linearly with the chain length. The core 1s^2^ orbitals of the carbon atoms are not correlated. In Figure 2, we report total timings of the calculations, as well timings for two major parts, namely, (i) evaluation of the  functional, and (ii) decomposition of the *T*_4_, *L*_3_, and *L*_4_ amplitudes. For clarity, the timings are given in relation to the calculations for methane. To confirm that the results given in Figure 2 match the theoretical predictions, we fitted the timings with the functional form *a*·*n*^*b*^ (a linear function on a logarithmic scale) for *n* = 3–8. We obtained the exponents *b* = 6.64 and *b* = 5.76 for the parts (i) and (ii), respectively. The empirically found values of the exponents are in both cases somewhat smaller than predicted theoretically (7 and 6). This can be explained by the fact that both parts of the calculations involve also many lower-scaling steps such as computation of intermediate quantities, and so forth. While the cost of such steps is asymptotically marginal, they still contribute non-negligibly to the total workload for systems that can be studied at present.

### 3.3 Calibration of the Method

In this section, we study errors of the proposed formalism in reproduction of the absolute (Q) correction. For this purpose, we selected a set of 16 small molecules comprising 2–5 first row atoms. Within the cc-pVTZ basis set employed in the calculations, the largest molecule is described by 118 atomic orbitals and hence the conventional CCSDT(Q) calculations are feasible with a reasonable computational cost. Therefore, the values of the exact (Q) correction are available for each molecule and shall be used as references in the present section. The list of molecular systems used in the benchmark calculations and their structures in Cartesian coordinates are provided in the Supporting Information. To assure that the error resulting from the density-fitting approximation does not contaminate the final conclusions of this section, a large cc-pV5Z-RI auxiliary basis set is used. We verified that this leads to errors of no larger than a few μ*H* in the correlation energies which is entirely negligible in the present context.

An important aspect of the analysis provided below relates to the determination of the recommended values of the quantities *N*_SVD_ and *N*_qua_ that serve as parameters in the rank-reduced formalism in the present work. The former parameter determines the size of the triple excitation subspaces used in *T*_3_ and *L*_3_ operators, see eq 2, while the latter serves the same purpose in the case of the quadruple excitation subspaces in *T*_4_ and *L*_4_. As the cost of the calculations increases steeply with the increase in *N*_SVD_ and *N*_qua_, it is necessary to recommend a way of determining these parameters for a given system such that a sufficient level of accuracy is attained while the computational cost is simultaneously minimized. Because both *N*_SVD_ and *N*_qua_ increase linearly with the system size, it is convenient to tie them to some quantity that shares the same property, but is known upfront for a given system. In this way, the parameters can be easily transferred between molecules of different sizes. Similarly to previous works, we express the parameters *N*_SVD_ and *N*_qua_ as fraction times the total number of active molecular orbitals in the system, *N*_MO_ (frozen-core orbitals and possibly frozen virtual orbitals are excluded). In other words, these parameters are given by *N*_SVD_ = *x*·*N*_MO_ and *N*_qua_ = *y*·*N*_MO_, where *x* and *y* are asymptotically independent of the system size. Note that both *x* and *y* may be larger than the unity. It has been shown in ref (14) that in reproduction of the CCSDT correlation energy, *N*_SVD_ ≈ *N*_MO_ is sufficient in usual applications. However, it cannot be guaranteed a priori that a similar size of the triple excitation subspace is adequate in the determination of the (Q) corrections.

**Figure 3:**
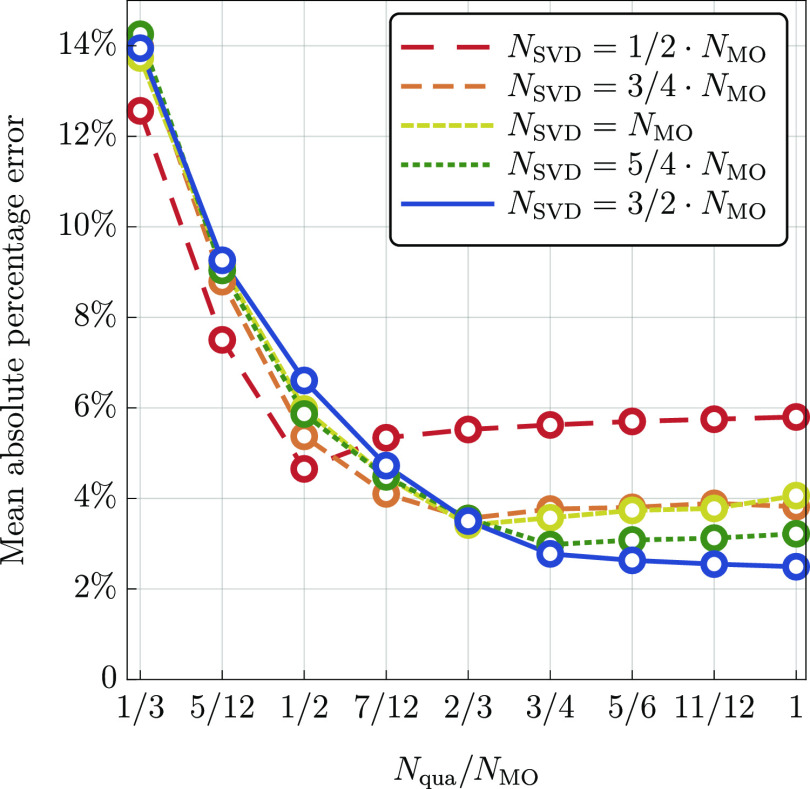
MAPE in the (Q) correction obtained using the  functional (cc-pVTZ basis set) as a function of *N*_qua_ for several representative values of the *N*_SVD_ parameter, see the legend. The exact CCSDT(Q) results are used as a reference. The symbol *N*_MO_ denotes the total number of orbitals in a given system.

Because the molecules included in the test set vary considerably in size, it is necessary to use a size-intensive error measure to compare the results. First, we consider the mean absolute percentage error (MAPE) averaged over all molecules. In Figure 3, we plot MAPE as a function of *N*_qua_ varied from 1/3*N*_MO_ to *N*_MO_ for several representative values of *N*_SVD_, namely, *N*_SVD_ = *x*·*N*_MO_ with *x* = 1/2, 3/4, 1, 5/4, 3/2. Overall, for each value of *N*_SVD_ individually, we observe a similar trend in error decay as *N*_qua_ is increased. Initially, the error vanishes rapidly, which is followed by a plateau region where the error stabilizes, see Figure 3. Beyond this point, further increase of the *N*_qua_ parameters leads to no appreciable accuracy improvements, and in some cases, the error even increases by a tiny amount. Clearly, in this region, the accuracy is limited by the error of the triply, and not quadruply excited, amplitudes. This is further confirmed by the observation that the error in the plateau region depends significantly on *N*_SVD_. For *N*_SVD_ = 1/2*N*_MO_, the error stabilizes at the level of around 6%; this decreases to around 4% for *N*_SVD_ = *N*_MO_ and to slightly above 2% for *N*_SVD_ = 3/2*N*_MO_.

To recommend values of the parameters *N*_SVD_ and *N*_qua_ that shall be used in future calculations, we require that the MAPE in Figure 3 should be at the order of a few percent according to the discussion in the Introduction. The smallest triple excitation subspace that systematically delivers the accuracy better than 5% corresponds to *N*_SVD_ = 3/4*N*_MO_ or *N*_SVD_ = *N*_MO_. Smaller values of *N*_SVD_ are not recommended, unless supported by some reference calculations that confirm their reliability or if larger errors are acceptable. Further increase, to about *N*_SVD_ = 3/2*N*_MO_, of the parameter *N*_SVD_ is necessary to reach accuracy levels of 2% or so. Regarding the value of the second parameter, it is desirable to set *N*_qua_ to a value that (for a given *N*_SVD_) corresponds as closely as possible to the onset of the plateau region. In this way, the computational cost of the procedure is minimized without compromising the accuracy. A conservative choice is to set *N*_qua_ = 2/3*N*_SVD_, such that for each *N*_SVD_ ≥ *N*_MO_, the value of *N*_qua_ lies well-within the plateau region. We recommend this setup in future calculations. This approach has one additional advantage: the values of *N*_SVD_ and *N*_qua_ do not have to be varied independently. Instead, they are increased simultaneously which makes the results easier to analyze and represent.

**Figure 4:**
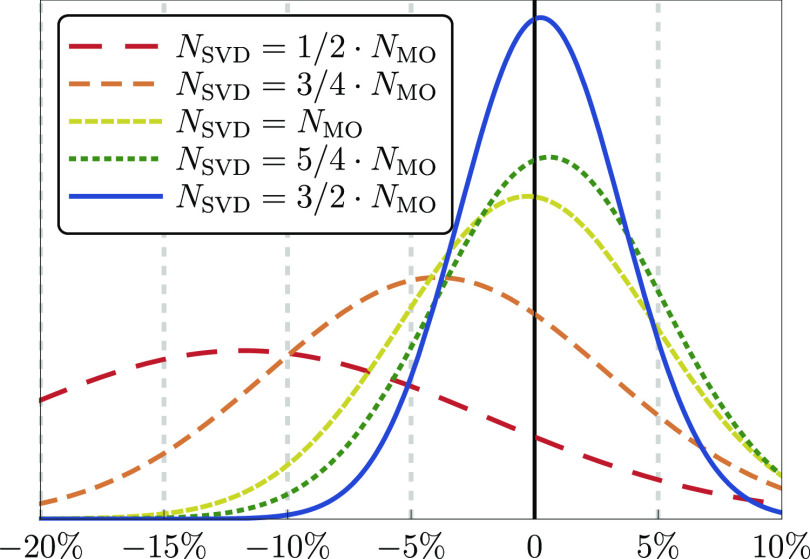
Distribution of relative errors (in percent) in the (Q) correction obtained using the  functional (cc-pVTZ basis set) for several representative values of the *N*_SVD_ parameter, see the legend. For each value of *N*_SVD_, the parameter *N*_qua_ is set to 2/3*N*_SVD_. The exact CCSDT(Q) results are used as a reference. The symbol *N*_MO_ denotes the total number of orbitals in a given system.

Thus far, we have concentrated on MAPE as a measure of error of the proposed formalism. While this error measure is particularly important from the practical point of view, it provides little information about the error distribution and its characteristics. To fill this gap, we consider signed percentage error defined as
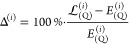
35where the index *i* enumerates the molecules in the test set, and *E*_(Q)_^(*i*)^ is the reference (exact) value of the (Q) correction for the *i*-th molecule. For the purposes of statistical analysis, we calculate the mean error, Δ, and its standard deviation, Δ_std_^2^ using the well-known formulas. For all cases considered here, we found that the error distribution is normal to a good degree of approximation. Therefore, for clarity, we represent the error measures Δ and Δ_std_ graphically in Figure 4 in terms of normalized Gaussian distributions. As illustrative examples, we consider *N*_SVD_ = *x*·*N*_MO_ with *x* = 1/2, 3/4, 1, 5/4, 3/2, and in each case, we set *N*_qua_ = 2/3*N*_SVD_ according to the discussion above. As seen in Figure 4, the accuracy obtained with *N*_SVD_ = 1/2·*N*_MO_ and *N*_SVD_ = 3/4·*N*_MO_ is not satisfactory. Although in the latter case the mean error is acceptable , the corresponding standard deviation is still large (Δ_std_ ≈ 6.8%) and hence the error distribution is rather broad. The results are improved considerably for *N*_SVD_ = *N*_MO_ and *N*_SVD_ = 5/4·*N*_MO_, where the mean error decreases to Δ ≈ −0.29% and Δ ≈ 0.59%, respectively. This is accompanied by a significant reduction of the standard deviation. Finally, by increasing the value of the parameter *N*_SVD_ to 3/2·*N*_MO_, the mean error is further reduced slightly , similarly as the standard deviation (Δ_std_ ≈ 3.3%).

Next, we consider errors of the proposed formalism in reproduction of relative energies. To this end, we prepared the following set of 10 chemical reactions:

1.,
2.,
3.,
4.,
5.,
6.,
7.,
8.,
9.,
10..

Contribution of the (Q) correction to each reaction energy has been computed with three different schemes (all within the cc-pVTZ orbital basis set). First, the exact CCSDT(Q) calculations that employ neither decomposition of the excitation amplitudes nor density-fitting approximation are used as a benchmark. The second and third schemes involve the quadratic (Q) functional with the recommended settings, namely, *N*_qua_ = 2/3*N*_SVD_, and for several representative *N*_SVD_. In the second scheme, we use a large cc-pV5Z-RI auxiliary basis set for density-fitting approximation and hence the DF errors are marginal. In the third scheme, the same settings are used, except that the standard cc-pVTZ-RI auxiliary basis set (matched to the used orbital cc-pVTZ) is employed. This enables us to study the impact of the density-fitting approximation on the quality of the results and establish whether the conventional auxiliary basis sets available in the literature are sufficient for the present purposes.

In Tables 2 and 3, we report results of the calculations with the large and standard density-fitting basis sets, respectively. As expected, for *N*_SVD_ = 1/2*N*_MO_ and *N*_SVD_ = 3/4*N*_MO_, average errors are substantial from the present point of view. However, as the size of the excitation subspace is increased to *N*_SVD_ = *N*_MO_, the average absolute errors decrease to about 0.1 kJ/mol. This confirms that the recommended settings perform well in the determination of the relative energies. Additionally, by comparing the results presented in Tables 2 and 3, one finds that the density-fitting approximation has a tiny impact on the quality of the results. The average DF errors are of the order of 0.01 kJ/mol and even the largest error found for the test set is well below 0.1 kJ/mol. Therefore, we conclude that the standard auxiliary basis sets (optimized for a given orbital basis) are sufficient for accurate determination of the (Q) correction.

**Table 2:** Contribution of the (Q) Correction to the Reaction Energies Calculated Using the Exact CCSDT(Q) Method and Using the Quadratic (Q) Functional as a Function of *N*_SVD_ (with Fixed *N*_qua_ = 2/3*N*_SVD_)^a^

		*N*_SVD_^b^				
reaction	exact	1/2*N*_MO_	3/4*N*_MO_	*N*_MO_	4/3*N*_MO_	3/2*N*_MO_
1	2.88	–0.12	–0.10	–0.12	–0.08	–0.06
2	1.52	–0.14	–0.20	–0.18	–0.10	–0.07
3	0.29	–0.12	0.02	0.06	0.02	0.02
4	1.61	–0.26	–0.10	0.04	0.01	0.01
5	3.15	–0.17	–0.06	0.12	0.13	0.10
6	–1.06	0.36	0.01	–0.17	–0.13	–0.05
7	–1.33	0.50	0.29	0.12	0.03	0.01
8	1.63	0.34	0.28	0.09	0.20	0.08
9	0.08	0.25	0.25	0.00	0.08	0.03
10	1.06	0.12	0.06	0.05	0.04	0.02
MAE		0.24	0.14	0.10	0.08	0.07
STD		0.27	0.17	0.12	0.10	0.07

a The mean absolute error (MAE) and the standard deviation of the error (STD) are given in the last two rows. The cc-pVTZ orbital basis set is used together with a large cc-pV5Z-RI auxiliary basis set to eliminate the error due to the density-fitting approximation. All values are given in kJ/mol.

b Errors (for a given *N*_SVD_) with respect to the exact result.

**Table 3:** Same Data as in Table 2 but Obtained Using the Standard cc-pVTZ-RI Auxiliary Basis Set for the Density-Fitting Approximation

		*N*_SVD_^a^				
reaction	exact	1/2*N*_MO_	3/4*N*_MO_	*N*_MO_	4/3*N*_MO_	3/2*N*_MO_
1	2.88	–0.16	–0.09	–0.06	–0.12	–0.06
2	1.52	–0.14	–0.29	–0.18	–0.11	–0.07
3	0.29	–0.11	0.02	0.08	0.03	0.03
4	1.61	–0.25	–0.14	0.05	0.01	0.09
5	3.15	–0.18	–0.08	0.13	0.13	0.10
6	–1.06	0.24	–0.17	–0.17	–0.12	–0.05
7	–1.33	0.39	–0.06	0.14	0.04	0.03
8	1.63	0.31	0.29	0.07	0.20	0.08
9	0.08	0.25	0.22	0.00	0.07	0.03
10	1.06	0.12	–0.20	0.04	0.03	0.02
MAE		0.21	0.16	0.09	0.08	0.07
STD		0.24	0.18	0.11	0.11	0.07

a Errors (for a given *N*_SVD_) with respect to the exact result.

It must be pointed out that with the parameters *N*_SVD_ and *N*_qua_ set as fixed multiples of some system-dependent quantity, the proposed method is not guaranteed to be size-consistent. This is true even one selects a multiple of a quantity that scales linearly with the system size, such as *N*_MO_ suggested in the previous paragraphs. While the results presented in this work, for example, for the reaction energies, show that the size-inconsistency error is tiny, this can still become problematic in applications, for example, to weakly interacting systems, where proper cancellation of non-physical size-inconsistent contributions is important. One may argue from a pragmatic standpoint that if significant size-inconsistency errors are encountered, the simplest remedy is to increase the constant factor that relates *N*_SVD_ and *N*_qua_ to *N*_MO_. As the rank-reduced formalism is built upon CC methods which are rigorously size-extensive, this approach is, in principle, always able to decrease these errors to acceptable levels. However, this line of reasoning is not fully satisfactory as it may lead to a significant increase of the computational costs. A more suitable approach would be to determine the size of the excitation subspace adaptively for a given molecule based on some numerical threshold that is transferable between systems. However, this problem is non-trivial as the HOOI algorithm applied to higher-order amplitudes does not provide a natural numerical parameter that can be used for the truncation, in contrast to, for example, the diagonalization approach adopted in ref (12) for the doubly excited amplitudes. Our preliminary tests have shown that a simple threshold on, for example, the eigenvalues of the *M*_*ai*,*bj*_ matrix defined by eq 27 are not entirely satisfactory. Therefore, a more elaborate scheme is required which employs, for example, a threshold on the cumulative eigenvalues of the *M*_*ai*,*bj*_ matrix. A complete analysis of this problem requires numerous benchmarks calculations analogous to the data presented in ref (81). This is beyond the scope of the present paper and requires a separate study which is currently in progress.

Finally, after establishing the computational protocol that shall be used in subsequent applications, we can assess the computational performance of the proposed theory in comparison with the conventional (Q) implementation. While we have shown in the previous section that the rank-reduced formalism is characterized by a lower scaling with the system size than the exact (Q) method (*N*^7^ vs *N*^9^), it is not yet clear how this translates into computational advantages. In fact, due to the overhead related to the determination of the excitation subspace (and other steps of the rank-reduced calculations), one expects that the prefactor of the conventional (Q) algorithm is lower and hence there is a break-even point beyond which our method is favored. In order to approximately locate this break-even point, we compared the timings of the calculations reported in this section with the conventional (Q) algorithm as implemented in the CFOUR program (to allow a fair comparison, the same machine was used for both calculations and serial program execution was requested). Depending on the cardinal number of the basis set, the proposed algorithm becomes beneficial for systems with more than 100–150 basis set functions. For example, for the formic acid molecule in the cc-pVTZ basis set (115 active orbitals), the rank-reduced calculations are three times faster than the conventional algorithm. In general, our tests revealed that the break-even point occurs faster for smaller basis sets. However, we also would like to point out that the current pilot implementation of the rank-reduced formalism can be improved by more extensive code optimization. While, in principle, the same is true for the conventional (Q) algorithm, implementations of the latter are much more mature.

### 3.4 Isomerization Energy of *ortho*/*meta* Benzyne

As the first application of the proposed theory, we study the isomers of the benzyne molecule (C_6_H_4_) in the singlet spin state. We are interested in the isomerization energy between *ortho*- and *meta*-benzyne. Benzynes have attracted a significant interest in recent years, both from experimental^82,83^ and theoretical points of view^84−91^ due to their unique electronic structure and chemical properties. In synthetic organic chemistry, *ortho*-benzyne is a crucial intermediate in several important types of reactions; see the recent review paper of Tadross and Stoltz^92^ for an in-depth discussion. Benzynes are also found^93^ to be the key intermediate in the formation of polycyclic aromatic hydrocarbons—carcinogenic and environmentally harmful compounds. In particular, the *ortho*- and *meta*-benzyne isomerization has been proposed to constitute an important step of various fragmentation and decomposition reaction pathways.^94−96^ From the theoretical standpoint, benzynes are known to possess a singlet diradical character of the ground-state energy level and it has been shown that static correlation effects play an important role in description of these systems. Therefore, benzynes are frequently employed in benchmark studies of novel quantum chemistry methods where accurate and reliable reference data are valuable.

Throughout the present section, we adopt the convention that the isomerization energy Δ*E* is defined as
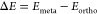
36It is known that in the ground electronic singlet state, the ortho isomer is more stable and hence the total isomerization energy defined above is positive. However, individual contributions to the isomerization energy representing various physical contributions may be of an arbitrary sign. For clarity, positive contributions are understood to favor the ortho isomer, while negative contributions—the meta isomer.

Besides studying the performance of the rank-reduced formalism for the isomers of benzyne, our goal is to show how the proposed method can be incorporated in the so-called composite electronic structure schemes in order to increase their accuracy, computational performance, or range of applicability. Several families of composite schemes were proposed in the literature, for example Gaussian-*n* (G-*n*) originally introduced by Pople et al.,^97−100^ Weizmann-*n* (W-*n*) model chemistry developed by Martin and collaborators,^101−103^ or HEATxyz protocol in its several variants.^104−106^ Nowadays, composite schemes are an important tool in, for example, ab initio thermochemistry calculations or theoretical prediction of quantitative chemical kinetics. The main idea behind the composite schemes is to split, for example, the total electronic energy of a molecule, into a sum of one (in some variants, two) major components supplemented by a series of smaller additive corrections. Depending on the desired level of accuracy, the number of corrections varies from just a few to a dozen or so in the most demanding situations. As the corrections are small in absolute terms compared to the major components, they can be calculated less accurately—usually employing a smaller basis set or with some additional approximations. Below we introduce a composite method that targets the accuracy comparable to the exact CCSDT(Q) theory and employs the rank-reduced approach to the calculation of the (Q) correction and the SVD-CCSDT+ method for determination of the triple excitation contributions.

The molecular geometries of *ortho*- and *meta*-benzyne were taken from the paper of Karton et al.^90^ where they were optimized using the frozen-core CCSD(T)/cc-pVQZ method. Our preliminary study has shown that these structures are well-converged with respect to the basis set size and the CC level. Therefore, all subsequent calculations of contributions to the isomerization energy were performed at fixed CCSD(T)/cc-pVQZ geometries, unless explicitly stated otherwise.

We begin by considering the Hartree–Fock contribution to the isomerization energy which was calculated using the cc-pV*X*Z basis sets with *X* = D, T, Q, 5. As expected, it converges very fast to the basis set limit, with the value calculated using the cc-pVQZ basis set, 106.90 kJ/mol, differs from the result obtained within the cc-pV5Z basis set, 106.97 kJ/mol, by just 0.07 kJ/mol. To further minimize the basis set incompleteness error, we perform three-parameter extrapolation using the exponential formula
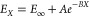
37where *E*_∞_, *A*, and *B* are fitted to reproduce the results obtained within the largest three basis sets. This leads to the final result 106.99 kJ/mol. Taking into account the rapid convergence of the results with the basis set size, it is reasonable to assume that the error of this quantity is no larger than 0.05 kJ/mol.

Next, we move on to the valence CCSD contribution using the same basis sets as for the Hartree–Fock method. One obtains −37.08, −34.90, −33.52, and −32.99 kJ/mol with the *X* = D, T, Q, 5 basis sets, respectively. The convergence pattern to the basis set limit is systematic, but the residual basis set error is still relatively large. To eliminate a significant portion of this error we employ the two-parameter Riemann extrapolation formula proposed in ref (107)
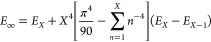
38This stencil is used for extrapolation of all correlation energies in the remainder of the present work. The extrapolated CCSD contribution using the *X* = Q(4), 5 basis sets reads −32.32 ± 0.34 kJ/mol, where the error was conservatively estimated to be equal to the half of the difference between the extrapolated value and the result in the largest basis set available. This approach was found to provide reliable and conservative error estimates for small molecular systems at the same level of theory.^107,108^

**Figure 5:**
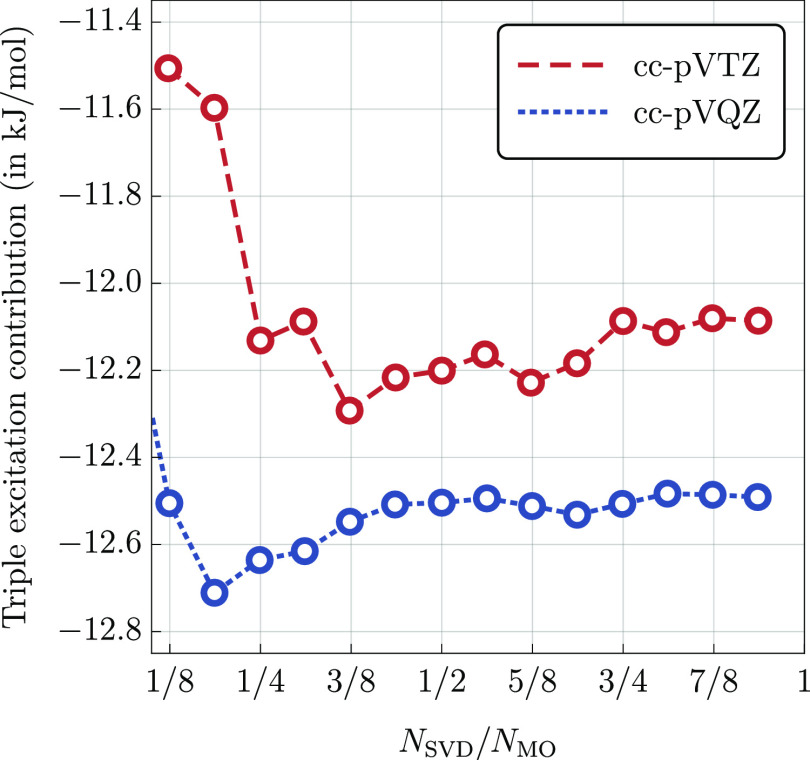
Triple excitation contribution to the isomerization energy of *ortho*/*meta* benzyne calculated using the SVD-CCSDT+ method (cc-pVTZ and cc-pVQZ basis sets) as a function of the *N*_SVD_ parameter. The symbol *N*_MO_ denotes the total number of orbitals in the system.

The next important contribution to the isomerization energy is the effect of triple excitations, defined as the difference of the results obtained with the CCSDT and CCSD methods. Due to cost considerations, it is a common practice to split the triple excitations contribution into two parts, namely, (i) the contribution of triple excitations captured by the CCSD(T) method and (ii) the remainder, that is, the difference between the CCSD(T) and CCSDT results. This approach is justified by the fact that the first contribution is usually dominating and can be obtained with a larger basis set. However, in the present application, we found this separation to be no longer beneficial if the SVD–CCSDT+ method is used for the evaluation of the triple excitation effects. This is because the largest basis set we could use in both CCSD(T) and SVD–CCSDT+ calculations was cc-pVQZ. Despite significant effort, it was impossible to perform canonical (T)/cc-pV5Z calculation using the hardware and software available to us, either due to excessive computational time or memory/disk space limitations. Taking into consideration that SVD–CCSDT+ is more accurate, we use it directly to determine the effects of triple excitations, bypassing the (T) method. In Figure 5, we show the convergence of the *T*_3_ contribution (cc-pVTZ and cc-pVQZ basis sets) to the isomerization energy as a function of the *N*_SVD_ parameter. Similarly as in the previous sections, this parameter is expressed as *N*_SVD_ = *x*·*N*_MO_, where *x* ∈ (0, 1]. The results are remarkably stable with respect to the value of *N*_SVD_; for *x* > 1/2, the results change by less than 0.1 kJ/mol with the smaller basis and 0.05 kJ/mol with the larger basis. We take the results obtained with *x* = 1 as the limit which leads to 12.12 kJ/mol and 12.48 kJ/mol within cc-pVTZ and cc-pVQZ basis sets, respectively. We assign the uncertainty of 0.05 kJ/mol to both these values. To obtain the final value of the triple excitation contribution to the isomerization energy, we perform two-point *X* = T, Q complete basis set extrapolation, giving 12.80 ± 0.17 kJ/mol. Two sources of error contribute to the proposed uncertainty: the extrapolation error (0.16 kJ/mol) which was estimated in the same way as for the CCSD contribution, and the error due to the truncation of the triple excitation subspace (0.05 kJ/mol, see the discussion above related to the *N*_SVD_ parameter). Because both sources of error can be viewed as independent, the final error is calculated by summing their squares and taking the square root according to the usual rules of error propagation.

**Figure 6:**
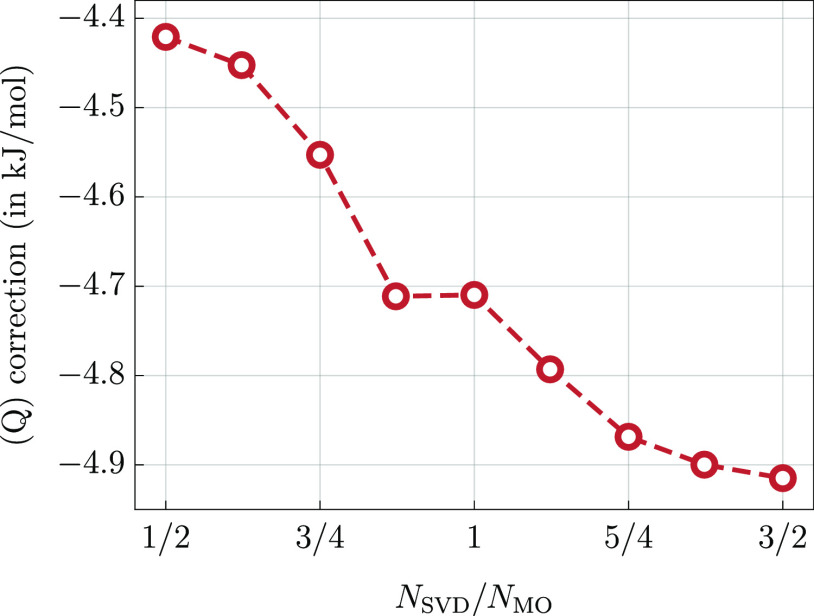
Quadruple excitation contribution to the isomerization energy of *ortho*/*meta*-benzyne calculated using the  functional (cc-pVTZ basis set) as a function of the *N*_SVD_ parameter. For each value of *N*_SVD_, the parameter *N*_qua_ is set to 2/3*N*_SVD_. The symbol *N*_MO_ denotes the total number of orbitals in the system.

Finally, we move on to the calculation of the (Q) correction which accounts for the quadruple excitation effects. For this purpose, we adopt the quadratic functional formalism introduced in the present work combined with the cc-pVTZ basis set. We also follow the recommendations stated in the previous section and set *N*_qua_ = 2/3*N*_SVD_ in all calculations. In Figure 6, we present the (Q) contribution to the isomerization energy as a function of the *N*_SVD_ parameter. Beyond *N*_SVD_ = *N*_MO_ changes in the (Q) correction are increasingly smaller. For example, upon increasing this parameter from *N*_SVD_ = *N*_MO_ to *N*_SVD_ = 5/4*N*_MO_, the (Q) correction decreases by about 0.15 kJ/mol, while further increase to *N*_SVD_ = 3/2*N*_MO_ affects it only by ca. 0.04 kJ/mol. By following the trend seen in Figure 6 one can expect that by further increase of the *N*_SVD_ parameter the (Q) correction will still decrease slightly. However, the changes are expected to be insignificant; even assuming the worst case scenario that the convergence of the (Q) correction is inversely proportional to *N*_SVD_, the limit would be less than 0.1 kJ/mol away from the value obtained with *N*_SVD_ = 3/2*N*_MO_. As a result, we assume that the (Q) correction to the isomerization energy is equal to the value obtained for *N*_SVD_ = 3/2*N*_MO_, and assign conservative 0.1 kJ/mol error bars, giving −4.92 ± 0.10 kJ/mol. We neglect the basis set incompleteness error in calculation of the (Q) correction. The computations of the (Q) correction using the quadratic functional for benzyne molecule with *N*_SVD_ = 3/2*N*_MO_ and 2/3*N*_SVD_ (cc-pVTZ basis set) take about 2 days on 14 cores of AMD Opteron Processor 6174.

The last major contribution to the isomerization energy is the zero-point vibration energy (ZPVE). Unfortunately, computation of this quantity at the CC level is costly, especially if a large basis set is required. For this reason, we employ the B3LYP/cc-pVTZ method to determine the ZPVE correction to the isomerization energy. Within the harmonic oscillator approximation, the ZPVE contribution equals to −4.34 kJ/mol. This value is further scaled by the recommended factor *f* = 0.9764 to take the anharmonic effects into account,^109^ giving −4.24 kJ/mol. In order to estimate the error of this quantity, we note that in a recent work, the B3LYP/cc-pVTZ method^110−113^ was found to perform extremely well in comparison with CCSD(T) for molecules composed of first-row atoms.^114^ This is especially true for hydrocarbons, where the average deviation from CCSD(T) is just about 1%. Therefore, we conservatively assume that the error of the ZPVE contribution to the isomerization energy of *ortho*/*meta* benzyne does not exceed 5%, or 0.21 kJ/mol.

Finally, we consider several minor corrections that do not contribute significantly to the isomerization energy, but are nonetheless required in an accurate study. In the order of importance, we consider first the effect of the inner-shell 1s^2^ orbitals of carbon atoms on the isomerization energy. The inner shell correction was computed as a difference between all-electron and frozen-core CCSD(T) results obtained within the core–valence cc-pwCV*X*Z basis sets.^117^ In this way, one obtains 0.64, 1.60, and 1.81 kJ/mol for *X* = D, T, Q, respectively. Our final estimation, 2.00 ± 0.10 kJ/mol, is obtained by two-point extrapolation from the *X* = T, Q pair, and the error is estimated in the same fashion as for the valence CCSD contribution.

The scalar relativistic effects were taken into account using DKH Hamiltonian^118−120^ as implemented in NWChem program.^121^ The relativistic correction was calculated at the all-electron CCSD(T)/cc-pwCV*X*Z level of theory, giving −0.23, −0.19, and −0.22 for *X* = D, T, Q, respectively. The final result, −0.26 ± 0.02 kJ/mol, was obtained using the same procedure as for the inner-shell correction. Lastly, the diagonal Born–Oppenheimer correction (DBOC, also known as the adiabatic correction in the literature) was calculated using the CCSD/cc-pVDZ method^122^ employing the CFOUR program. The result, equal to about 0.03 kJ/mol, signals that this effect has a negligible impact on the isomerization energy. While the size of the basis set used is small, and the obtained value is only a rough estimation, it is sufficient for the present purposes. However, we assign large error bars to this quantity, 0.03 ± 0.10 kJ/mol.

The results obtained in this section are summarized in Table 4 and compared with other data available in the literature. The comparison with the most recent experimental determination^83^ reveals a substantial difference of about 10 kJ/mol. However, it has to be pointed out that the experimental value was obtained as a combination of atomization energies and the error of the final result is difficult to estimate. We find it likely that the theoretical value obtained in this work is considerably more accurate which is supported by other theoretical results found in the literature. They all tend to cluster around Δ*E* ≈ 50–55 kJ/mol which suggest that the experimental value should be revised down.

**Table 4:** Final Error Budget of the Calculations of the Isomerization Energy of *ortho*/*meta* Benzyne^a^

	contribution to Δ*E*
Hartree–Fock	106.99 ± 0.05
valence CCSD	–32.32 ± 0.34
valence *T*_3_	–12.80 ± 0.17
valence (Q)	–4.92 ± 0.10
inner-shell correlation	2.00 ± 0.10
scalar relativity	–0.26 ± 0.04
DBOC	0.03 ± 0.10
ZPVE	–4.24 ± 0.21
total	54.52 ± 0.47
experiment	64.0^83,115^
other theoretical	54.4^b^^90^
	51.5^c^^91^
	61.2^d^^87^
	51.0^e^^84^

a All values are given in kJ/mol.

b W3.2lite(b) composite method.

c CAS(12,12) + PT2/CBS + ZPE_CASSCF_.

d G2M(rcc,MP2) composite method.^116^

e CASPT2[g1] + aANO/C(5s4p2d)/H(3s2p) basis set.

### 3.5 Cope Rearrangement in Bullvalene Molecule

**Figure 7:**
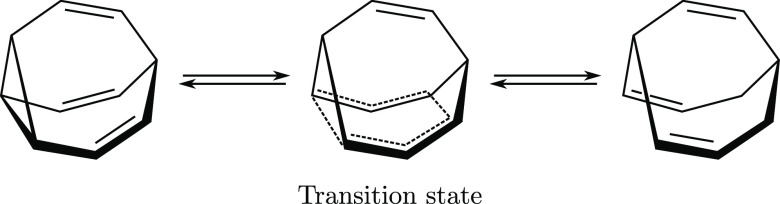
Chemical structures illustrating the Cope rearrangement in the bullvalene molecule.

The second system we study in this work in detail is the bullvalene molecule, C_10_H_10_. This molecule attracted considerable attention because it is a prototypical fluxional molecule that possesses no permanent molecular structure, that is, the nuclei are constantly in a concerted motion.^123^ In bullvalene, this is enabled by the Cope rearrangement, exemplified in Figure 7, that may occur between many equivalent configurations. The initial and final structure are degenerate, but are separated by a reaction barrier. While the bullvalene molecule has been synthesized a long time ago^124,125^ and frequently studied both experimentally and theoretically since then, the height of the barrier is not established unambiguously. The most recent theoretical result of Karton et al.^126^ differs from the experimental results (obtained by NMR techniques^127^) by several kJ/mol. This discrepancy is much larger than the reported uncertainties of both calculations and measurements, and hence the theory and experimental data are not consistent at this point. In this section, we carry an independent systematic theoretical study of the bullvalene Cope rearrangement barrier height and discuss the possible sources of this inconsistency. In particular, we include corrections due to triple and quadruple excitations calculated with the rank-reduced formalism. These corrections would be extremely costly to compute using the exact CCSDT(Q) method; in fact, we did not manage to accomplish CCSDT(Q) calculations even with the smallest cc-pVDZ basis.

The electronic contribution to the reaction barrier height is denoted by the symbol Δ*E*^‡^. For the purposes of direct comparison with the experimental data, we additionally need to calculate the Gibbs free energy barrier heights at the temperature *T* = 298 K. This quantity is denoted by Δ*G*_298_^‡^ and includes, besides Δ*E*^‡^, the zero-point vibrational energy (ZPVE) and enthalpic/entropic temperature corrections, as detailed below.

The molecular geometries of the bullvalene equilibrium structure and Cope rearrangement transition state were optimized at the B3LYP-D3/pc-2 level of theory^128−131^ using the NWChem package. The obtained structures were verified to represent the equilibrium structure (real harmonic frequencies) and first-order transition state (one imaginary frequency). The Cartesian geometries of both structures are given in the Supporting Information. The barrier height Δ*E*^‡^ is split into several components calculated at different levels of theory, and a composite scheme is used to assemble the best theoretical estimate. Because the bullvalene molecule is roughly twice as large as the systems considered in Section 3.4, the composite method applied here is less rigorous in nature. In particular, we do not assign uncertainties to individual contributions to the barrier height; instead, we attach a global error estimate only to the final result.

First, we consider the Hartree–Fock contribution to the barrier height which was calculated using the cc-pV*X*Z basis sets with *X* = T, Q, 5. The exponential extrapolation (37) from these three basis sets leads to the result 108.97 kJ/mol. This differs by less than 0.1 kJ/mol from the result obtained within the cc-pV5Z basis, showing that the error of the Hartree–Fock component of Δ*E*^‡^ is negligible. The second contribution to Δ*E*^‡^ was calculated using the CCSD method, giving −29.20, −28.39, and −27.50 kJ/mol with cc-pV*X*Z, *X* = D, T, Q, basis sets, respectively. To further reduce the basis set incompleteness error, we apply the two-point extrapolation formula 38 resulting in the final CCSD contribution of −26.68 kJ/mol.

**Figure 8:**
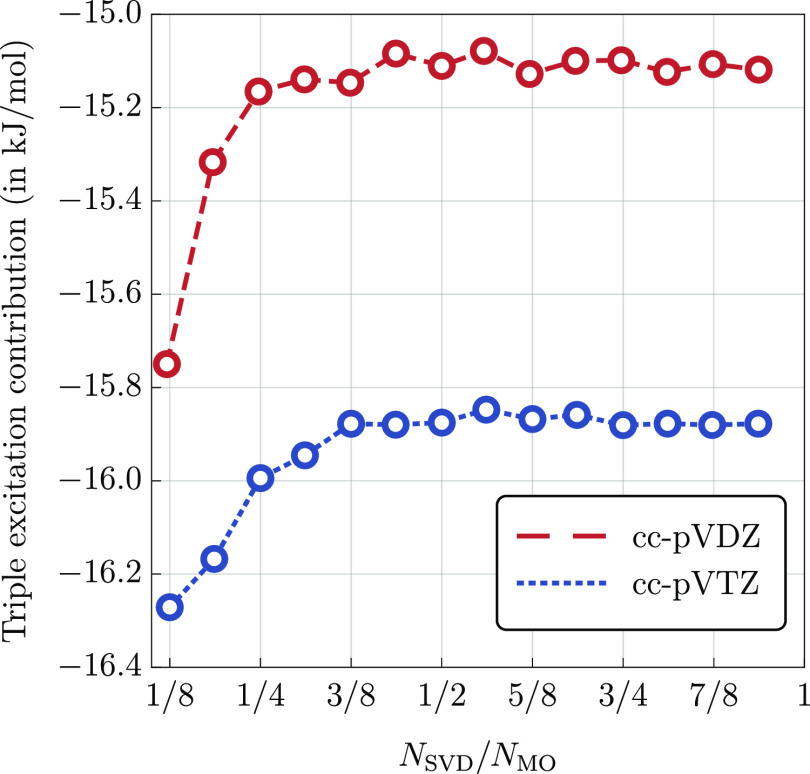
Triple excitation contribution to the Cope rearrangement barrier height (Δ*E*^‡^) in the bullvalene molecule calculated using the SVD-CCSDT+ method (cc-pVDZ and cc-pVTZ basis sets) as a function of the *N*_SVD_ parameter. The symbol *N*_MO_ denotes the total number of orbitals in the system.

Next, we consider the contribution of triple excitations to the barrier height. It was computed using the SVD–CCSDT+ method within the cc-pVDZ and cc-pVTZ basis sets. Similarly as in the previous section, we do not split the effect of triple excitations into (T) and post-(T) components because we did not manage to calculate the (T) correction within a larger (cc-pVQZ) basis set due to excessive time requirements. Note that the SVD–CCSDT+ calculations within the cc-pVTZ basis involve 50 correlated electrons and 440 atomic orbitals which vastly exceed the capabilities of the available CCSDT implementations. In Figure 8, we present triple excitation contribution to the barrier height as a function of the *N*_SVD_ parameter. The results saturate fast with respect to the value of *N*_SVD_, and for *N*_SVD_ = 1/2*N*_MO_, they are essentially converged. Beyond this point, minor fluctuations at the level of ca. 0.05 and 0.02 kJ/mol are observed, but this is completely negligible in comparison with other sources of error. Using the results obtained with *N*_SVD_ = *N*_MO_, we obtain the contributions of triple excitations equal to −15.23 and −15.87 kJ/mol in the cc-pVDZ and cc-pVTZ basis sets, respectively. The final result, −16.26 kJ/mol, is obtained using the two-point extrapolation formula, eq 38.

**Figure 9:**
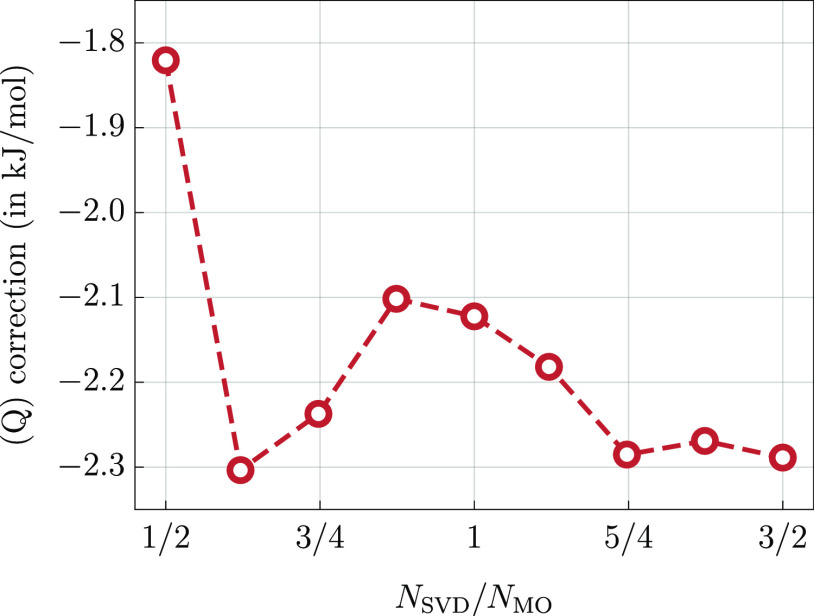
Quadruple excitation contribution to the Cope rearrangement barrier height (Δ*E*^‡^) in the bullvalene molecule calculated using the  functional (cc-pVDZ basis set) as a function of the *N*_SVD_ parameter. For each value of *N*_SVD_, the parameter *N*_qua_ is set to 2/3*N*_SVD_. The symbol *N*_MO_ denotes the total number of orbitals in the system.

The quadruple excitation contribution to Δ*E*^‡^ was calculated using the  functional and the cc-pVDZ basis set. In Figure 9, we present the (Q) correction as a function of the *N*_SVD_ parameter and with the recommended *N*_qua_ = 2/3*N*_SVD_. One can see that beyond *N*_SVD_ = 5/4*N*_MO_, the results are essentially stable with respect to this parameter. The variations are within 0.01–0.02 kJ/mol and hence are negligible from the present point of view. Therefore, we take the value obtained with *N*_SVD_ = 3/2*N*_MO_, namely, −2.29 kJ/mol, as the final contribution of quadruple excitations to Δ*E*^‡^. The computations of the (Q) correction using the quadratic functional for the bullvalene molecule with *N*_SVD_ = 5/4*N*_MO_ and 2/3*N*_SVD_ (cc-pVDZ basis set) take about 3 days on 14 cores of AMD Opteron Processor 6174.

The last contributions to Δ*E*^‡^ are due to the inner-shell correlation and relativistic effects (scalar DKH Hamiltonian). They were both calculated using the all-electron CCSD method within the cc-pwCVTZ basis set supplemented by (T) correction obtained within cc-pwCVDZ basis. No extrapolation toward the complete basis set was performed. This brings contributions to Δ*E*^‡^ equal 1.33 and −0.21 kJ/mol due to the aforementioned two effects. We also estimated the DBOC component of Δ*E*^‡^ (CCSD/cc-pVDZ level of theory) and found it to be negligible (<0.1 kJ/mol) within the present accuracy standards.

Finally, ZPVE contribution to the barrier height, as well as thermal corrections, was calculated at the same level of theory as the geometry optimization (B3LYP-D3/pc-2). The raw value of ZPVE was additionally scaled by the empirical factor *f* = 0.9678, as recommended in ref (132), to take the anharmonic effects into account, giving −4.36 kJ/mol. Thermal corrections were calculated within the rigid rotor/harmonic oscillator approximations without frequencies scaling. The thermal enthalpic and entropic contributions to the Gibbs free energy barrier height for *T* = 298 K are −0.59 and 1.03 kJ/mol, respectively, and hence the total finite-temperature correction is just 0.44 kJ/mol.

The final results of the calculations of the Gibbs free energy barrier height for the Cope rearrangement in the bullvalene molecule are summarized in Table 5. The total Δ*G*_298_^‡^ determined by us equals to 60.94 kJ/mol. In order to roughly estimate the error of this result, we note that there are two major sources of uncertainty: valence CCSD and ZPVE contributions. They can both lead to errors of the order of 0.5 kJ/mol. The remaining contributions to Δ*G*_298_^‡^ are expected to be accurate to within 0.1–0.2 kJ/mol. All in all, the value determined by us, Δ*G*_298_^‡^ = 60.94 kJ/mol, has an uncertainty of around 1 kJ/mol. This result is in reasonable agreement with the most recent theoretical result of Karton,^126^ 62.21 kJ/mol, but in a disagreement with older calculations based on lower levels of theory which give results within 35–55 kJ/mol range.^133−136^ More strikingly, our result is in a disagreement with the experimental data of Moreno et al.^127^ who obtained Δ*G*_298_^‡^ = 54.8 ± 0.8 kJ/mol from gas-phase NMR measurements. Such a large difference of about 6 kJ/mol is unlikely to be caused by an error in the theoretical protocol adopted by us. Therefore, we believe that the experimental data for this system should be reevaluated and a new measurement may help to resolve the persisting discrepancy between state-of-the-art theory and experimental results.

**Table 5:** Summary of the Calculations of the Gibbs Free Energy Barrier Height for the Cope Rearrangement in the Bullvalene Molecule^a^

	contribution to Δ*G*_298_^‡^
Hartree–Fock	108.97
valence CCSD	–26.68
valence *T*_3_	–16.26
valence (Q)	–2.29
inner-shell correlation	1.33
scalar relativity	–0.21
ZPVE	–4.36
thermal correction	0.44
total	60.94

a All values are given in kJ/mol.

## 4 Conclusions and Future Work

In this work, we have extended the rank-reduced CC formalism to the calculation of non-iterative energy corrections due to quadruple excitations. The focus of the present work has been concentrated on the CCSDT(Q) method, which has become the de facto standard in high-accuracy ab initio quantum chemistry, and can be viewed as the “platinum standard” of the field. The proposed formalism consists of two major novel components. The first is the application of the Tucker format to compress the quadruple excitation amplitudes and eliminate the full rank *t*_*ijkl*_^*abcd*^ tensor entirely from the computational procedure. The second is the introduction of a modified functional for evaluation of the (Q) correction. This functional is rigorously equivalent to the standard (Q) formalism when the exact CC amplitudes are used. However, due to the fact that the new functional is stationary with respect to the amplitudes, it is less susceptible to errors resulting from the aforementioned compression. We show, both theoretically and numerically, that the computational cost of the proposed method scales as the seventh power of the system size. Using reference results for a set of small molecules, the method is calibrated to deliver accuracy of a few percent in relative energies. To illustrate the potential of the theory, we calculate the isomerization energy of *ortho*/*meta* benzyne (C_6_H_4_) and the barrier height for the Cope rearrangement in bullvalene (C_10_H_10_). In both cases, we show that the proposed formalism considerably increases the range of applicability of the CC theory with non-iterative energy corrections due to quadruple excitations.

The present work is a starting point for a rank-reduced treatment of other quantum chemistry methods involving quadruple excitations. Indeed, the quadruple excitation subspace obtained by the HOOI procedure, Section 2.5, can be used also in more advanced (both iterative and non-iterative) CC models involving the *T*_4_ operator. This includes even the complete CCSDTQ method. In fact, our preliminary study showed that the *N*^7^ scaling can be achieved at the CCSDTQ level if both the triple and quadruple excitation amplitudes are compressed using the Tucker format. However, to exploit this advantage, an efficient implementation is required to minimize the prefactor, and the accuracy of the resulting method must be thoroughly tested and calibrated.

Another important extension is generalization of the rank-reduced CC formalism to the open-shell situations. This direction is especially important for applications in ab initio thermochemistry, where calculation of atomization energies is an important problem. A straightforward way to handle the open-shell systems is offered by the spin-unrestricted CC theory, but this approach leads to the spin-contamination of the electronic wave function, as is well-documented in the literature.^137−139^ While the issue of spin-contamination may not be severe in many applications, a more pressing problem is the need to handle numerous spin cases of the triply and, especially, quadruply excited configurations. In the spin-unrestricted formalism, each spin case has to be decomposed separately, leading to a significant increase in the computational costs. As a result, a more robust and advanced^140−146^ approach to the spin-adaptation in open-shell systems may be required which will be considered in future works.

## Supporting Information Available

Derivation of the error formula for the L(Q) functional; approximate treatment of the *L*_4_, *T*_4_, and *L*_*3*_ operators; factorization of the working expressions; computational protocol; impact of the approximations introduced in the quadratic functional; comparison of computational timings; errors in absolute energies with standard auxiliary basis sets; optimized structures of bullvalene; and optimized molecular geometries from Section 3.3 in the *.xyz format (PDF)
