# Supplementary material for: When Gold Is Not Enough: Platinum Standard of Quantum Chemistry with N7 Cost
Source: J Chem Theory Comput. 2022 Oct 31;18(11):6537–56. doi: 10.1021/acs.jctc.2c00460 (PMC9648181; doi:10.1021/acs.jctc.2c00460)
Supplement: Supplementary file 1 — ct2c00460_si_001.pdf [file ct2c00460_si_001.pdf]

Supporting Information

When gold is not enough: platinum standard of quantum chemistry with N^7 cost

Michał Lesiuk*

Faculty of Chemistry, University of Warsaw

Pasteura 1, 02-093 Warsaw, Poland

E-mail: m.lesiuk@uw.edu.pl

Contents

SI.1 Notation used in the Supporting Information **3**

SI.2 Derivation of the error formula for the $\mathcal{L}_{(\mathrm{Q})}$ functional **3**

SI.3 Approximate treatment of the L_4 operator **5**

SI.4 Approximate treatment of the T_4 operator **7**

SI.5 Approximate treatment of the L_3 operator **9**

SI.6 Factorization of the working expression for $\langle T_2|[W,T_4]\rangle$ **10**

SI.7 Factorization of the working expression for $\langle T_3|[W,T_4]\rangle$ **11**

SI.8 Factorization of the working expression for $\langle L_4|[F,T_4]\rangle$ **13**

SI.9 Evaluation of the $\langle L_3|e^{-T}He^{T}\rangle$ term **13**

SI.10 Factorization of the working expression for $\langle L_4|\Big[\big[W, T_2\big], T_2\Big]\rangle$ **14**

SI.11 Computational protocol **15**

SI.12 Impact of the approximations introduced in the quadratic functional **16**

SI.13 Molecules used in benchmark calculations in Sec. 3.3 **16**

SI.14 Comparison of computational timings **17**

SI.15 Errors in absolute energies with standard auxiliary basis sets **18**

SI.16 Optimized structures of bullvalene **19**

SI.1 Notation used in the Supporting Information

All equations in the Supporting Information are numbered according to the format SI.n, where $n = 1, 2, \ldots$, and are referenced within this document as Eq. (SI.n). The usual symbol Eq. (n) is reserved for referencing equations from the main text. The rest of the notation is the same as in the main text, with the exception of several additional quantities defined below.

SI.2 Derivation of the error formula for the $\mathcal{L}_{(\mathrm{Q})}$ functional

Denote the exact T_3, T_4, L_3, and L_4 amplitudes by the superscript "ex". For clarity, we recall that the operator T_3^{ex} is obtained from the standard CCSDT method, while T_4^{ex} is a result of solving Eq. (4) exactly. Similarly, L_3^{ex} and L_4^{ex} are obtained by solving Eqs. (11) and (12) without any approximations. Additionally, let us denote by $\mathcal{L}_{(\mathrm{Q})}^{\mathrm{ex}}$ the value of the functional (9) evaluated with the exact amplitudes; according to the discussion from Sec. 2.3, it is equal to the sum of Eqs. (7) and (8). In actual calculations we are in possession of only some approximate counterparts, which we denote by the superscript "app", i.e. T_3^{app}, T_4^{app}, L_3^{app}, and L_4^{app}. The value of the functional (9) evaluated with the approximate amplitudes is likewise denoted by $\mathcal{L}_{(\mathrm{Q})}^{\mathrm{app}}$. Without any loss over generality, it is possible to rewrite the approximate amplitudes as a sum of the exact amplitudes and some error term, e.g. $T_3^{\mathrm{app}} = T_3^{\mathrm{ex}} + \delta T_3$. Similar formulas hold also for the remaining operators, giving rise to the corresponding error terms δL_3, δT_4, and δL_4. Moreover, the difference between the exact and approximate value of the (Q) functional is denoted by $\delta\mathcal{L}_{(\mathrm{Q})} = \mathcal{L}_{(\mathrm{Q})}^{\mathrm{app}} - \mathcal{L}_{(\mathrm{Q})}^{\mathrm{ex}}$. In order to prove that the second condition discussed in Sec. 2.3 is fulfilled by the proposed (Q) functional, it is sufficient to show that $\delta\mathcal{L}_{(\mathrm{Q})}$ can be expressed as a sum of terms which are all quadratic in the combined powers of δT_3, δL_3, δT_4, and δL_4. To demonstrate this property, we start

with the formal definition

$$\begin{aligned}\delta\mathcal{L}_{(\mathrm{Q})} &= \langle T_2|\big[W, T_4^{\mathrm{app}}\big]\rangle + \langle T_3^{\mathrm{app}}|\big[W, T_4^{\mathrm{app}}\big]\rangle + \langle L_3^{\mathrm{app}}|\, e^{-T^{\mathrm{app}}} H e^{T^{\mathrm{app}}}\rangle \\ &+ \langle L_4^{\mathrm{app}}|\big[F, T_4^{\mathrm{app}}\big] + \big[W, T_3^{\mathrm{app}}\big] + \frac{1}{2}\Big[\big[W, T_2\big], T_2\Big]\rangle - \mathcal{L}_{(\mathrm{Q})}^{\mathrm{ex}}.\end{aligned} \tag{SI.1}$$

where $T^{\mathrm{app}} = T_1 + T_2 + T_3^{\mathrm{app}}$. Next, we insert the definition $T_3^{\mathrm{app}} = T_3^{\mathrm{ex}} + \delta T_3$ (and analogous relationships for the remaining operators) into Eq. (9) and obtain after some rearrangements

$$\begin{aligned}\delta\mathcal{L}_{(\mathrm{Q})} &= \langle T_2|\big[W, T_4^{\mathrm{ex}}\big]\rangle + \langle T_2|\big[W, \delta T_4\big]\rangle + \langle T_3^{\mathrm{ex}}|\big[W, T_4^{\mathrm{ex}}\big]\rangle + \langle T_3^{\mathrm{ex}}|\big[W, \delta T_4\big]\rangle \\ &+ \langle \delta T_3|\big[W, T_4^{\mathrm{ex}}\big]\rangle + \langle \delta T_3|\big[W, \delta T_4\big]\rangle + \langle L_3^{\mathrm{ex}} + \delta L_3|\, e^{-T^{\mathrm{ex}}-\delta T_3} H e^{T^{\mathrm{ex}}+\delta T_3}\rangle \\ &+ \langle L_4^{\mathrm{ex}} + \delta L_4|\big[F, T_4^{\mathrm{ex}}\big] + \big[W, T_3^{\mathrm{ex}}\big] + \frac{1}{2}\Big[\big[W, T_2\big], T_2\Big]\rangle \\ &+ \langle L_4^{\mathrm{ex}}|\big[F, \delta T_4\big] + \big[W, \delta T_3\big]\rangle + \langle \delta L_4|\big[F, \delta T_4\big] + \big[W, \delta T_3\big]\rangle - \mathcal{L}_{(\mathrm{Q})}^{\mathrm{ex}}.\end{aligned} \tag{SI.2}$$

Straight away, several simplifications are possible in this formula. First, we note that the first term on the right hand side is equal to $E_{\mathrm{Q}}^{[5]}$ defined by Eq. (SI.27), while the third is equal to $E_{\mathrm{Q}}^{[6]}$, cf. Eq. (8). Together, these two terms give the exact $E_{(\mathrm{Q})}$ correction which cancels the counter-term $-\mathcal{L}_{(\mathrm{Q})}^{\mathrm{ex}}$. Second, the eighth term on the right hand side vanishes, because the exact T_4^{ex} amplitudes obey Eq. (4). We are therefore left with

$$\begin{aligned}\delta\mathcal{L}_{(\mathrm{Q})} &= \langle T_2|\big[W, \delta T_4\big]\rangle + \langle T_3^{\mathrm{ex}}|\big[W, \delta T_4\big]\rangle + \langle \delta T_3|\big[W, T_4^{\mathrm{ex}}\big]\rangle + \langle \delta T_3|\big[W, \delta T_4\big]\rangle \\ &+ \langle L_3^{\mathrm{ex}} + \delta L_3|\, e^{-T^{\mathrm{ex}}-\delta T_3} H e^{T^{\mathrm{ex}}+\delta T_3}\rangle + \langle L_4^{\mathrm{ex}}|\big[F, \delta T_4\big]\rangle + \langle L_4^{\mathrm{ex}}|\big[W, \delta T_3\big]\rangle \\ &+ \langle \delta L_4|\big[F, \delta T_4\big] + \big[W, \delta T_3\big]\rangle.\end{aligned} \tag{SI.3}$$

Next, we note that the sum of the first, second and sixth terms is equal to zero, that is

$$\langle T_2|\big[W, \delta T_4\big]\rangle + \langle T_3^{\mathrm{ex}}|\big[W, \delta T_4\big]\rangle + \langle L_4^{\mathrm{ex}}|\big[F, \delta T_4\big]\rangle = 0, \tag{SI.4}$$

because the $L_4^{\rm ex}$ operator obeys the relationship (12). Once these three terms are eliminated the simplified formula reads

$$
\begin{aligned}
\delta\mathcal{L}_{(\mathrm{Q})} = &\langle \delta T_3 | \big[W, T_4^{\rm ex}\big] \rangle + \langle \delta T_3 | \big[W, \delta T_4\big] \rangle + \langle L_3^{\rm ex} + \delta L_3 | \, e^{-T^{\rm ex} - \delta T_3} H e^{T^{\rm ex} + \delta T_3} \rangle \\
&+ \langle L_4^{\rm ex} | \big[W, \delta T_3\big] \rangle + \langle \delta L_4 | \big[F, \delta T_4\big] + \big[W, \delta T_3\big] \rangle .
\end{aligned}
\tag{SI.5}
$$

Let us now consider the third term on the right hand side of the above expression. By using the nested commutator expansion (BCH expansion) one obtains

$$
\langle L_3^{\rm ex} + \delta L_3 | \, e^{-T^{\rm ex} - \delta T_3} H e^{T^{\rm ex} + \delta T_3} \rangle = \langle L_3^{\rm ex} + \delta L_3 | \left[e^{-T^{\rm ex}} H e^{T^{\rm ex}}, \delta T_3 \right] \rangle . \tag{SI.6}
$$

Higher-order commutators vanish because of conflicting excitation levels, while the zeroth-order term, i.e. $\langle L_3^{\rm ex} + \delta L_3 | \, e^{-T^{\rm ex}} H e^{T^{\rm ex}} \rangle$, is zero due to the CCSDT stationary conditions. Upon inserting back the above formula into Eq. (SI.5) we additionally take into account the following condition

$$
\langle \delta T_3 | \big[W, T_4^{\rm ex}\big] \rangle + \langle L_4^{\rm ex} | \big[W, \delta T_3\big] \rangle + \langle L_3^{\rm ex} | \left[e^{-T^{\rm ex}} H e^{T^{\rm ex}}, \delta T_3 \right] \rangle = 0, \tag{SI.7}
$$

which is a special case of Eq. (11). This allows to bring Eq. (SI.5) into the following form

$$
\delta\mathcal{L}_{(\mathrm{Q})} = \langle \delta T_3 | \big[W, \delta T_4\big] \rangle + \langle \delta L_3 | \left[e^{-T^{\rm ex}} H e^{T^{\rm ex}}, \delta T_3 \right] \rangle + \langle \delta L_4 | \big[F, \delta T_4\big] + \big[W, \delta T_3\big] \rangle , \tag{SI.8}
$$

which coincides with Eq. (13) from the main text.

SI.3 Approximate treatment of the L_4 operator

The starting point is Eq. (23) in which the Laplace transformation of energy denominators, Eq. (32), is additionally used. The resulting equation can be explicitly written in terms of

T_2 amplitudes and two-electron integrals as follows

$$l_{ijkl}^{abcd} = \frac{1}{4} \sum_{g}^{N_g} w_g \, e^{-t_g \left(\epsilon_i^a + \epsilon_j^b + \epsilon_k^c + \epsilon_l^d\right)} P_{ijkl}^{abcd} \Big[(ai|bj) \, t_{kl}^{cd} \Big], \tag{SI.9}$$

where the permutation operator P_{ijkl}^{abcd} has the same meaning as in Eq. (35). The HOOI procedure applied to l_{ijkl}^{abcd} necessitates evaluation of the following partly-contracted quantity

$$\lambda_{ai,BCD} = l_{ijkl}^{abcd} \, V_{bj}^{B} \, V_{ck}^{C} \, V_{dl}^{D} \, . \tag{SI.10}$$

By recalling Eq. (SI.9) it can be rewritten as

$$\lambda_{ai,BCD} = \sum_{g}^{N_g} w_g \, e^{-t_g \left(\epsilon_i^a + \epsilon_j^b\right)} \left(1 + P_{BD} + P_{BC}\right) \Big[(ai|bj) \, V_{bj}^{B} \, t_{CD}^{g} + t_{ij}^{ab} \, V_{bj}^{B} \, I_{CD}^{g} \Big], \tag{SI.11}$$

where P_{AB} is a transposition operator that exchanges the indices A and B, while

$$t_{CD}^{g} = t_{kl}^{cd} \, e^{-t_g \left(\epsilon_k^c + \epsilon_l^d\right)} \, V_{ck}^{C} \, V_{dl}^{D} \, , \tag{SI.12}$$

$$I_{CD}^{g} = (ai|bj) \, e^{-t_g \left(\epsilon_i^a + \epsilon_j^b\right)} \, V_{ai}^{C} \, V_{bj}^{D} \, . \tag{SI.13}$$

Note that the above quantities are symmetric with respect to the exchange of the two lower indices. Where the basis vectors V_{ai}^{A} are determined, the core tensor l_{ABCD} is found from an explicit formula

$$l_{ABCD} = \sum_{g}^{N_g} w_g \left(1 + P_{BD} + P_{BC}\right) \Big[I_{AB}^{g} \, t_{CD}^{g} + I_{CD}^{g} \, t_{AB}^{g} \Big]. \tag{SI.14}$$

The computational cost of evaluating (SI.10) and Eq. (SI.14) scales as $O^2 V^2 N_{\text{qua}} \propto N^5$.

SI.4 Approximate treatment of the T_4 operator

For efficiency sake, in evaluation of the quantity $\gamma^g_{ai,BCD}$ we introduce the second approximation besides the Laplace transform of the energy denominators, namely the eigendecomposition of the doubly-excited SVD-CCSDT amplitudes

$$t^{ab}_{ij} = W^F_{ai}\, s_F\, W^F_{bj}. \tag{SI.15}$$

The benefit of employing this formula is that all eigenvectors corresponding to eigenvalues below a predefined threshold, i.e., $|s_F| < \delta$, can be excluded from the summation with a marginal impact on the accuracy. Moreover, for a fixed value of δ the number of remaining eigenvalues (referred to as N_{neig} further in the text) scales linearly with the system size, i.e. $N_{\text{neig}} \propto N$. Adoption of this approximation does not reduce the scaling of the procedure used to compute $\gamma^g_{ai,BCD}$, but it significantly reduces the prefactor and simplifies the working expressions. The cost of obtaining the decomposition (SI.15) is proportional to $O^3V^3 \propto N^6$ and hence it is acceptable from the present point of view. The quantities W^F_{ai} and s_F have to be obtained only once at the end of the SVD-CCSDT calculations and do not change during the iterative part of the HOOI procedure.

To express the quantity $\gamma^g_{ai,BCD}$ in a compact form we introduce a handful of intermediate quantities. The first group of intermediates is independent on the $\hat{V}^{Ag}_{ai}$ vectors and hence they can be calculated before the HOOI procedure is initiated

$$B^{QX}_{ai} = B^Q_{ae}\, U^X_{ei} - B^Q_{mi}\, U^X_{am}, \quad \bar{B}^{QF}_{ai} = \Big(B^Q_{ae}\, W^F_{ei} - B^Q_{mi}\, W^F_{am}\Big) s_F. \tag{SI.16}$$

With help of the these intermediates, the quantity Γ^{abcd}_{ijkl} defined in Eq. (17) is rewritten as

$$\Gamma^{abcd}_{ijkl} = \frac{1}{2} P^{abcd}_{ijkl} \Big[B^Q_{ai}\, B^{QX}_{bj}\, U^Y_{ck}\, U^Z_{dl}\, t_{XYZ} - 2(ai|me)\, t^{eb}_{kj}\, t^{cd}_{ml} + \bar{B}^{QF}_{ai}\, \bar{B}^{QG}_{bj}\, W^F_{dl}\, W^G_{ck} \Big]. \tag{SI.17}$$

The remaining intermediates change during every iteration

$$\alpha^g_{XA} = U^X_{ai}\,\hat{V}^{Ag}_{ai}, \quad \bar{\alpha}^g_{FA} = W^F_{ai}\,\hat{V}^{Ag}_{ai}, \quad \beta^g_{QA} = B^Q_{ai}\,\hat{V}^{Ag}_{ai}, \tag{SI.18}$$

$$\zeta^{Ag}_{ai} = W^F_{ai}\,\bar{\alpha}^g_{FA}, \quad \omega^g_{XAQ} = B^{QX}_{ai}\,\hat{V}^{Ag}_{ai}, \quad \bar{\omega}^g_{FAQ} = \bar{B}^{QF}_{ai}\,\hat{V}^{Ag}_{ai}. \tag{SI.19}$$

All of the intermediates defined above can be computed with the cost proportional to N^5 or less. The last two objects (ω^g_{XAQ} and $\bar{\omega}^g_{FAQ}$) are typically the most expensive to calculate, scaling as $OVN_{\text{aux}}N_{\text{neig}}N_{\text{qua}}$. In our implementation the intermediate quantities are stored on the disk and read in batches with one of the indices fixed whenever required. This eliminates the need to store them in memory in full.

By exploiting the intermediates defined above, we are now in a position to present the factorized formula

$$\begin{aligned}
\gamma^g_{ai,BCD} = P_{BCD}\Big[& \underbrace{\frac{1}{2}\Big(B^Q_{ai}\,\omega^g_{XCQ} + B^{QX}_{ai}\beta^g_{QC}\Big)\left(t_{XYZ}\,\alpha^g_{YB}\,\alpha^g_{ZD}\right)}_{OVN_{\text{aux}}N^3_{\text{qua}}\propto N^6} + \underbrace{\zeta^{Dg}_{am}\Big(\zeta^{Cg}_{ei}\big(B^Q_{me}\,\beta^g_{QB}\big)\Big)}_{O^2VN^3_{\text{qua}}\propto N^6} \\
& + \underbrace{U^Y_{ai}\left(\omega^g_{XCQ}\,\beta^g_{QB}\right)\left(t_{XYZ}\,\alpha^g_{ZD}\right)}_{OVN_{\text{SVD}}N^3_{\text{qua}}\propto N^6} + \underbrace{\Big(\bar{B}^{QG}_{ai}\,\bar{\alpha}^g_{GD}\Big)\left(\bar{\omega}^g_{FBQ}\,\bar{\alpha}^g_{FC}\right)}_{OVN_{\text{aux}}N^3_{\text{qua}}\propto N^6} \\
& + \underbrace{W^F_{ai}\Big(\bar{\omega}^g_{FCQ}\left(\bar{\alpha}^g_{GB}\,\bar{\omega}^g_{GDQ}\right)\Big)}_{OVN_{\text{neig}}N^3_{\text{qua}}\propto N^6} + \underbrace{B^Q_{ai}\,\hat{V}^{Dg}_{dl}\Big(\zeta^{Cg}_{dm}\big(B^Q_{me}\,\zeta^{Bg}_{el}\big)\Big)}_{OVN_{\text{aux}}N^3_{\text{qua}}\propto N^6} \\
& + \underbrace{W^F_{ai}\Big(\zeta^{Cg}_{el}\big(\beta^g_{QB}\,B^Q_{me}\big)\Big)\big(\hat{V}^{Dg}_{dl}\,W^F_{dm}\big)}_{OVN_{\text{neig}}N^3_{\text{qua}}\propto N^6} + \underbrace{W^F_{ai}\Big(W^F_{el}\big(\beta^g_{QB}\,B^Q_{me}\big)\Big)\big(\hat{V}^{Dg}_{dl}\,\zeta^{Cg}_{dm}\big)}_{OVN_{\text{neig}}N^3_{\text{qua}}\propto N^6}\Big],
\end{aligned} \tag{SI.20}$$

$P_{BCD} = (1 + P_{BD})\,(1 + P_{BC} + P_{DC})$. Below each term we provide the scaling of the rate-determining contraction step. Overall, the cost of evaluating Eq. (SI.20) is asymptotically dominated by the first, fourth and sixth terms, assuming that $N_{\text{aux}} \gg N_{\text{SVD}}, N_{\text{qua}}$ which is always true with the recommended settings of the control parameters and with the standard density-fitting basis sets. Note that in the above formula there are no terms that scale as

N^7 or worse, proving the statements from the main text.

SI.5 Approximate treatment of the L_3 operator

To apply the HOOI procedure to the l_{ijk}^{abc} amplitudes, we need a robust expression for the product $l_{ai,Y'Z'} = l_{ijk}^{abc}\, U_{bj}^{Y'}\, U_{ck}^{Z'}$. The approximate amplitudes are given by the explicit expression, see Eq. (28):

$$l_{ijk}^{abc} \approx (\epsilon_{ijk}^{abc})^{-1} \langle \mu_3 | \big[W, L_4\big] \rangle = (\epsilon_{ijk}^{abc})^{-1} \gamma_{ijk}^{abc}, \tag{SI.21}$$

where

$$\gamma_{ijk}^{abc} = P_{ijk}^{abc} \Big[I_{ia}^{B'C'} V_{bj}^{B'} V_{ck}^{C'} - B_{ia}^{QA'}\, B_{lk}^{QC'}\, l_{A'B'C'D'}\, V_{bj}^{B'} V_{cl}^{D'} \Big], \tag{SI.22}$$

with

$$B_{ia}^{QA'} = B_{mi}^{Q}\, V_{am}^{A'} - B_{ad}^{Q}\, V_{di}^{A'}, \tag{SI.23}$$

$$B_{lk}^{QC'} = B_{le}^{Q}\, V_{ek}^{C'}, \tag{SI.24}$$

$$I_{ia}^{B'C'} = \Big[B_{ia}^{QA'} \big(B_{le}^{Q}\, V_{el}^{D'} \big) + \frac{1}{2} \big(B_{ad}^{Q}\, V_{dl}^{D'} \big) \big(B_{le}^{Q}\, V_{ei}^{A'} \big) + \frac{1}{2} \big(B_{le}^{Q}\, V_{al}^{D'} \big) \big(B_{mi}^{Q}\, V_{em}^{A'} \big) \Big] l_{A'B'C'D'} \tag{SI.25}$$

The cost of computing these intermediates in $\propto N^5$ if the decomposition of the $l_{A'B'C'D'}$ amplitudes described in Sec. SI.3 is exploited. The projected quantity $l_{ai,Y'Z'}$ is now expressed

as

$$
\begin{aligned}
l_{ai,Y'Z'} = \big(1 + P_{Y'Z'}\big)\Big[& I_{ia}^{B'C'}\,\big(V_{bj}^{B'}\,U_{bj}^{Y'}\big)\big(V_{ck}^{C'}\,U_{ck}^{Z'}\big) \\
& + V_{ai}^{B'}\,\big(I_{jb}^{B'C'}\,U_{bj}^{Y'}\big)\big(V_{ck}^{C'}\,U_{ck}^{Z'}\big) \\
& + V_{ai}^{C'}\,\big(I_{kc}^{B'C'}\,U_{ck}^{Z'}\big)\big(V_{bj}^{B'}\,U_{bj}^{Y'}\big) \\
& - \underbrace{B_{ia}^{QA'}\,\big(V_{bj}^{B'}\,U_{bj}^{Y'}\big)\Big[\Big(B_{lk}^{QC'}\,\big(V_{cl}^{D'}\,U_{ck}^{Z'}\big)\Big)\,l_{A'B'C'D'}\Big]}_{OVN_{\mathrm{aux}}N_{\mathrm{SVD}}^{2}N_{\mathrm{qua}}} \\
& - \underbrace{\big(B_{jb}^{QA'}\,U_{bj}^{Y'}\big)\,V_{ai}^{B'}\Big[\Big(B_{lk}^{QC'}\,\big(V_{cl}^{D'}\,U_{ck}^{Z'}\big)\Big)\,l_{A'B'C'D'}\Big]}_{N_{\mathrm{aux}}N_{\mathrm{SVD}}N_{\mathrm{qua}}^{3}} \\
& - \underbrace{\Big[\Big(\big(B_{kc}^{QA'}\,U_{ck}^{Z'}\big)\,B_{li}^{QC'}\Big)\,l_{A'B'C'D'}\Big]V_{al}^{D'}\,\big(V_{bj}^{B'}\,U_{bj}^{Y'}\big)}_{O^{2}N_{\mathrm{SVD}}N_{\mathrm{qua}}^{3}}\Big]
\end{aligned}
\tag{SI.26}
$$

The scaling of the first three terms is N^5, while for the remaining ones is given in the underbraces [it is assumed that Eq. (SI.14) is exploited].

SI.6 Factorization of the working expression for $\langle T_2|\big[W, T_4\big]\rangle$

Below we provide explicit expression for the $\langle T_2|\big[W, T_4\big]\rangle$ correction in a factorized form. The computational cost of the rate-determining contraction step is given below each individual term. The notation for all quantities is the same as in the main text. Note that in the computation of $\langle T_2|\big[W, T_4\big]\rangle$ we do not exploit the factorization (SI.15), i.e. the "exact"

doubly-excited amplitudes are used below.

$$
\begin{aligned}
\langle T_2|\big[W,T_4\big]\rangle = & \underbrace{V^{A}_{ak}\,V^{C}_{ci}\Big[B^{Q}_{ai}\Big[\bar{t}^{cd}_{kl}\,\Big(\big(t_{ABCD}\,V^{B}_{bl}\,V^{D}_{dj}\big)\,B^{Q}_{bj}\Big)\Big]\Big]}_{O^2V^2N_{\mathrm{aux}}N^2_{\mathrm{qua}}\propto N^7} \\
& - 2\underbrace{\Big[\Big[(bi||aj)\,V^{B}_{bi}\Big]\,V^{A}_{ak}\Big]t_{ABCD}\Big[\Big(\bar{t}^{cd}_{lk}\,V^{C}_{cl}\Big)\,V^{D}_{dj}\Big]}_{O^2N^4_{\mathrm{qua}}\propto N^6} \\
& + \underbrace{\Big[(ai||bj)\,V^{A}_{ai}\,V^{B}_{bj}\Big]t_{ABCD}\Big[\bar{t}^{cd}_{kl}\,V^{C}_{ck}\,V^{D}_{dj}\Big]}_{O^2V^2N_{\mathrm{qua}}\propto N^5}.
\end{aligned}
\tag{SI.27}
$$

SI.7 Factorization of the working expression for $\langle T_3|\big[W,T_4\big]\rangle$

Similarly as in the previous section, in the computation of $\langle T_3|\big[W,T_4\big]\rangle$ we do not exploit the factorization (SI.15). To represent the $\langle T_3|\big[W,T_4\big]\rangle$ correction in a concise form we first introduce two intermediates

$$
A^{X}_{ld,kc} = \big(2 - P_{cd}\big)(ld|ki)\,U^{X}_{ci} - \big(2 - P_{kl}\big)(ld|ce)\,U^{X}_{ek}, \tag{SI.28}
$$

$$
\widetilde{A}^{X}_{ld,kc} = \big(1 + P_{ld,kc}\big)A^{X}_{ld,kc}, \tag{SI.29}
$$

where the operator P_{ij} exchanges the indices i and j, and $P_{ld,kc}$ is defined in the main text. Explicit formula for the $\langle T_3|\big[W,T_4\big]\rangle$ correction with the triply-excited amplitudes tensor t^{abc}_{ijk} decomposed according to Eq. (2) reads

$$
\begin{aligned}
\langle T_3|\big[W,T_4\big]\rangle = 2\,t_{XYZ}\,U^{Y}_{bj}\,U^{Z}_{ai}\Big[& - T^{klij}_{abcd}\,A^{X}_{ld,kc} + 2\,T^{kjli}_{abcd}\,\widetilde{A}^{X}_{lc,kd} \\
& - T^{kilj}_{abcd}\,\widetilde{A}^{X}_{lc,kd} - 2\,T^{ijkl}_{abcd}\,A^{X}_{ld,kc} + T^{jikl}_{abcd}\,A^{X}_{ld,kc}\Big].
\end{aligned}
\tag{SI.30}
$$

The last four terms in the above formula can be immediately factorized by exploiting the rank-reduced form of the quadruply-excited amplitudes tensor, see Eq. (8). This gives

$$\begin{aligned}
T^{kjli}_{abcd}\,\widetilde{A}^X_{lc,kd}\,t_{XYZ}\,U^Y_{bj}\,U^Z_{ai} &= \Big(\big(V^B_{bj}\,U^Y_{bj}\,t_{XYZ}\big)U^Z_{ai}\Big)\Big[\Big(\big(V^C_{cl}\,\widetilde{A}^X_{lc,kd}\big)V^D_{di}\Big)t_{ABCD}\Big]V^A_{ak},\\
T^{kilj}_{abcd}\,\widetilde{A}^X_{lc,kd}\,t_{XYZ}\,U^Y_{bj}\,U^Z_{ai} &= \Big[\Big[\Big(\big(t_{XYZ}\,U^Y_{bj}\big)V^B_{bi}\Big)U^Z_{ai}\Big]V^A_{ak}\Big]\Big(\big(\widetilde{A}^X_{lc,kd}\,V^C_{cl}\big)V^D_{dj}\Big)t_{ABCD},\\
T^{ijkl}_{abcd}\,A^X_{ld,kc}\,t_{XYZ}\,U^Y_{bj}\,U^Z_{ai} &= \Big(\big(A^X_{ld,kc}\,V^C_{ck}\,V^D_{dl}\big)\,t_{ABCD}\Big)\Big[\big(V^A_{ai}\,U^Z_{ai}\big)\big(V^B_{bj}\,U^Y_{bj}\big)t_{XYZ}\Big],\\
T^{ijkl}_{abcd}\,A^X_{ld,kc}\,t_{XYZ}\,U^Y_{bj}\,U^Z_{ai} &= \Big(\big(A^X_{ld,kc}\,V^C_{ck}\,V^D_{dl}\big)\,t_{ABCD}\Big)\Big[\Big(\big(V^B_{bi}\,U^Y_{bj}\big)U^Z_{ai}\Big)t_{XYZ}\Big]V^A_{aj}.
\end{aligned} \tag{SI.31}$$

The scaling of the first two terms is $O^2 N_{\mathrm{SVD}} N_{\mathrm{qua}}^4 \propto N^7$ in the rate-limiting step, while the third and fourth term are less expensive, $O^2 V^2 N_{\mathrm{SVD}} N_{\mathrm{qua}} \propto N^6$ and $OVN_{\mathrm{qua}} N_{\mathrm{SVD}}^3 \propto N^6$, respectively. The first term in Eq. (SI.30) is more problematic and to achieve a satisfactory factorization we first rewrite it as

$$-2\,t_{XYZ}\,U^Y_{bj}\,U^Z_{ai}\,T^{klij}_{abcd}\,A^X_{ld,kc} = 2\,t_{XYZ}\,U^Y_{bj}\,U^Z_{ai}\,T^{klij}_{abcd}\left[2B^Q_{ld}\,B^{QX}_{ck} - B^Q_{lc}\,B^{QX}_{dk}\right], \tag{SI.32}$$

where the quantities B^{QX}_{ck} are defined in Eq. (SI.16). To achieve this form one has to exploit the permutational symmetry of the quadruply-excited amplitudes. Both sub-expressions in the above formula are now factorized to the form (numerical prefactors are omitted for brevity)

$$t_{XYZ}\,U^Y_{bj}\,U^Z_{ai}\,T^{klij}_{abcd}\,B^Q_{ld}\,B^{QX}_{ck} = \Big[\big(U^Y_{bj}\,V^B_{bl}\big)\Big[\Big(t_{XYZ}\,\big(U^Z_{ai}\,V^A_{ak}\big)\Big)\big(B^{QX}_{ck}\,V^C_{ci}\big)\Big]\Big]\big(B^Q_{ld}\,V^D_{dj}\big)t_{ABCD}, \tag{SI.33}$$

$$t_{XYZ}\,U^Y_{bj}\,U^Z_{ai}\,T^{klij}_{abcd}\,B^Q_{lc}\,B^{QX}_{dk} = \Big[\big(U^Y_{bj}\,V^B_{bl}\big)\Big[\Big(t_{XYZ}\,\big(U^Z_{ai}\,V^A_{ak}\big)\Big)\big(B^{QX}_{dk}\,V^D_{dj}\big)\Big]\Big]\big(B^Q_{lc}\,V^C_{ci}\big)t_{ABCD}, \tag{SI.34}$$

which permits their evaluation with scaling $O^2 N_{\mathrm{aux}} N_{\mathrm{SVD}}^2 N_{\mathrm{qua}}^2 \propto N^7$ in the rate-limiting steps.

SI.8 Factorization of the working expression for $\langle L_4|\big[F,T_4\big]\rangle$

The term $\langle L_4|\big[F,T_4\big]\rangle$ written explicitly consists of five components:

$$\langle L_4|\big[F,T_4\big]\rangle = t_{ijkl}^{abcd}\,\epsilon_{ijkl}^{abcd}\bigg[-\frac{2}{3}\,l_{ijkl}^{abcd} + 2l_{ijkl}^{abdc} - \frac{4}{3}l_{ijkl}^{adbc} - \frac{1}{2}l_{ijkl}^{badc} + \frac{1}{2}l_{ijkl}^{dabc}\bigg]. \tag{SI.35}$$

By inserting Eqs. (21) and (24) and defining the intermediates

$$E_{AA'} = V_{ai}^{A}\,\epsilon_i^a\,V_{ai}^{A'},\quad S_{AA'} = V_{ai}^{A}\,V_{ai}^{A'}, \tag{SI.36}$$

$$M_{ij}^{AA'} = V_{ai}^{A}\,V_{aj}^{A'},\quad N_{ij}^{AA'} = V_{ai}^{A}\,\epsilon_i^a\,V_{aj}^{A'}. \tag{SI.37}$$

we arrive at

$$\begin{aligned}\langle L_4|\big[F,T_4\big]\rangle = t_{ABCD}\,l_{A'B'C'D'}\bigg[&\underbrace{-\frac{8}{3}\,E_{AA'}\,S_{BB'}\,S_{CC'}\,S_{DD'}}_{N_{\text{qua}}^5} + \underbrace{4\,E_{AA'}\,S_{BB'}\,M_{kl}^{CD'}\,M_{lk}^{DC'}}_{N_{\text{qua}}^6}\\ &+ \underbrace{4\,S_{AA'}\,S_{BB'}\,N_{kl}^{CD'}\,M_{lk}^{DC'}}_{N_{\text{qua}}^6} - \underbrace{\frac{4}{3}\,E_{AA'}\,M_{jk}^{BC'}\,M_{kl}^{CD'}\,M_{lj}^{DB'}}_{O^2N_{\text{qua}}^5} - \underbrace{4\,S_{AA'}\,N_{jk}^{BC'}\,M_{kl}^{CD'}\,M_{lj}^{DB'}}_{O^2N_{\text{qua}}^5}\\ &- \underbrace{2N_{ij}^{AB'}\,M_{ji}^{BA'}\,M_{kl}^{CD'}\,M_{lk}^{DC'}}_{N_{\text{qua}}^6} + \underbrace{2N_{ij}^{AB'}\,M_{jk}^{BC'}\,M_{kl}^{CD'}\,M_{li}^{DA'}}_{O^2N_{\text{qua}}^5}\bigg],\end{aligned} \tag{SI.38}$$

where the scaling of each term is given in the underbraces.

SI.9 Evaluation of the $\langle L_3|e^{-T}He^{T}\rangle$ term

Define

$$r_{ijk}^{abc} = \langle{}_{ijk}^{abc}|e^{-T}He^{T}\rangle. \tag{SI.39}$$

This quantity can be calculated with $O^2V^2N_{\mathrm{SVD}}^3$ cost as discussed in our previous work [J. Chem. Theory Comput. **17**, 7632 (2021)]. It is straightforward to show that

$$\langle L_3|e^{-T}He^{T}\rangle = \frac{1}{3}\, l_{ijk}^{abc} \Big(4r_{ijk}^{abc} - 6r_{ijk}^{acb} + 2r_{ijk}^{cab} \Big). \tag{SI.40}$$

To avoid storing the intermediate r_{ijk}^{abc} in memory, it is computed on-the-fly with a triple of indices (ijk) being fixed and immediately used in evaluation of $\langle L_3|e^{-T}He^{T}\rangle$. The second quantity l_{ijk}^{abc} with given (ijk) is reconstructed from the decomposed form (27).

SI.10 Factorization of the working expression for $\langle L_4|\Big[\big[W, T_2\big], T_2\Big]\rangle$

The explicit expression reads

$$\begin{aligned}
\langle L_4|\Big[\big[W, T_2\big], T_2\Big]\rangle =& - 4\, l_{ijkl}^{dcba}\, (mj|ni)\, \bar{T}_{mk}^{ab}\, \bar{T}_{nl}^{dc} + 2\, l_{ijkl}^{dbca}\, (mj|ni)\, T_{mk}^{ab}\, \bar{T}_{nl}^{dc} \\
& + 2\, l_{ijkl}^{dbca}\, (nj|mi)\, T_{mk}^{ab}\, \bar{T}_{nl}^{cd} - 2\, l_{ijkl}^{dbac}\, (mj||ni)\, T_{mk}^{ab}\, \bar{T}_{nl}^{dc} \\
& - 2\, l_{ijkl}^{dbca}\, (mj||ni)\, \bar{T}_{mk}^{ba}\, T_{nl}^{dc} + 4\, l_{ijkl}^{dbac}\, (mj||ni)\, \bar{T}_{mk}^{ba}\, T_{nl}^{dc} \\
& - 4\, l_{ijkl}^{dbac}\, (ae||mi)\, \bar{T}_{kj}^{eb}\, \bar{T}_{ml}^{dc} - 4\, l_{ijkl}^{adbc}\, (me||ai)\, \bar{T}_{kj}^{be}\, \bar{T}_{ml}^{dc} \\
& + 4\, l_{ijkl}^{adcb}\, (me||ai)\, \bar{T}_{kj}^{be}\, \bar{T}_{ml}^{dc} + 4\, l_{ijkl}^{dcba}\, (ae|mi)\, \bar{T}_{kj}^{be}\, \bar{T}_{ml}^{dc} \\
& + 4\, l_{ijkl}^{dcab}\, (ae|mi)\, \bar{T}_{kj}^{eb}\, \bar{T}_{ml}^{dc} + 4\, l_{ijkl}^{bdac}\, (ae|mi)\, \bar{T}_{kj}^{eb}\, \bar{T}_{ml}^{dc} \\
& - 4\, l_{ijkl}^{bdca}\, (ae|mi)\, \bar{T}_{kj}^{be}\, \bar{T}_{ml}^{cd} + 4\, l_{ijkl}^{dcba}\, (me|ai)\, \bar{T}_{kj}^{be}\, \bar{T}_{ml}^{cd} \\
& + 4\, l_{ijkl}^{bdac}\, (me|ai)\, \bar{T}_{kj}^{be}\, \bar{T}_{ml}^{dc} - 4\, l_{ijkl}^{dcab}\, (me|ai)\, T_{kj}^{be}\, \bar{T}_{ml}^{cd} \\
& - 4\, l_{ijkl}^{dcab}\, (me|ai)\, T_{kj}^{eb}\, \bar{T}_{ml}^{dc} - 4\, l_{ijkl}^{bdca}\, (me|ai)\, T_{kj}^{be}\, \bar{T}_{ml}^{dc} \\
& - 4\, l_{ijkl}^{dcba}\, (af|be)\, \bar{T}_{ji}^{ce}\, \bar{T}_{lk}^{fd} + 2\, l_{ijkl}^{dbca}\, (af|be)\, T_{ji}^{ce}\, \bar{T}_{lk}^{fd} \\
& + 2\, l_{ijkl}^{dbca}\, (af|be)\, T_{ji}^{ce}\, \bar{T}_{lk}^{df} - 2\, l_{ijkl}^{dbca}\, (af||be)\, \bar{T}_{ji}^{ec}\, T_{lk}^{fd} \\
& - 2\, l_{ijkl}^{cbda}\, (af||be)\, T_{ji}^{ce}\, \bar{T}_{lk}^{fd} + 4\, l_{ijkl}^{cbda}\, (af||be)\, \bar{T}_{ji}^{ec}\, T_{lk}^{fd}
\end{aligned} \tag{SI.41}$$

Further factorization of this formula is achieved by inserting Eqs. (SI.9) and (SI.14), leading to the cost of evaluating Eq. (SI.41) proportional to $O^3V^3N_{\text{qua}} \propto N^7$ in the rate liming step.

SI.11 Computational protocol

The scheme for computation of the (Q) correction consists of the following steps:

1. perform the HOOI of the l_{ijkl}^{abcd} amplitudes as described in Sec. SI.3 and evaluate the quantities I_{AB}^g, t_{CD}^g needed in Eq. (SI.14); the computational cost of this step is usually marginal;

2. perform HOOI of the l_{ijk}^{abc} amplitudes with the term $\langle\mu_3|\big[W,T_4\big]\rangle$ neglected as described in Sec. SI.5; do not compute the core matrix $l_{X',Y'Z'}$ yet ($OVN_{\text{aux}}N_{\text{qua}}N_{\text{SVD}}^2$ scaling);

3. calculate the terms $\langle L_4|\Big[W,T_3\Big]\rangle$ and $\langle L_4|\Big[\big[W,T_2\big],T_2\Big]\rangle$; the former term is analogous to $\langle T_3|\Big[W,T_4\Big]\rangle$ (see below), while the computation of the latter is discussed in Sec. SI.10 (scaling $O^2N_{\text{aux}}N_{\text{SVD}}^2N_{\text{qua}}^2 \propto N^7$ and $O^3V^3N_{\text{qua}} \propto N^7$, respectively);

4. perform HOOI of the t_{ijkl}^{abcd} amplitudes as described in Sec. SI.4 and compute the core tensor t_{ABCD} (scaling $O^2V^2N_{\text{qua}}^3 \propto N^7$);

5. compute $\langle T_2|\big[W,T_4\big]\rangle$, $\langle T_3|\big[W,T_4\big]\rangle$, and $\langle L_4|\big[F,T_4\big]\rangle$ terms as described in Secs. SI.6, SI.7, SI.8, respectively (scaling $O^2V^2N_{\text{aux}}N_{\text{qua}}^2 \propto N^7$, $O^2N_{\text{aux}}N_{\text{SVD}}^2N_{\text{qua}}^2 \propto N^7$, $O^2N_{\text{qua}}^5$);

6. evaluate the core $l_{X',Y'Z'}$ tensor with both $\langle\mu_{ijk}^{abc}|\big[W,T_4\big]\rangle$ and $\langle\mu_3|\big[W,L_4\big]\rangle$ terms included, see Sec. SI.5 (marginal cost);

7. calculate the $\langle L_3|e^{-T}He^T\rangle$ term, see Sec. SI.9 (scaling $O^2V^2N_{\text{SVD}}^3$).

SI.12 Impact of the approximations introduced in the quadratic functional

Table 1: Impact of the proposed approximations on the components of the (Q) correction evaluated using the quadratic functional. The results (in mH) are given for HF molecule.

term	cc-pVDZ	cc-pVTZ	cc-pVQZ
without approximations other than HOOI			
$\langle T_2\vert\big[W,T_4\big]\rangle$	−0.529	−0.128	−0.030
$\langle T_3\vert\big[W,T_4\big]\rangle$	−0.031	−0.165	−0.284
$\langle L_3\vert\, e^{-T}He^{T}\rangle$	−0.021	−0.069	−0.019
$\langle L_4\vert\big[F,T_4\big]\rangle$	−0.600	−0.284	−0.029
$\langle L_4\vert\big[W,T_3\big]\rangle$	0.182	0.575	0.826
$\frac{1}{2}\langle\Big[\big[W,T_2\big],T_2\Big]\rangle$	0.594	0.733	−0.813
approximations to L4: neglect of $\langle T_3\vert\big[W,\mu_4\big]\rangle$ from Eq. (12)			
$\langle T_2\vert\big[W,T_4\big]\rangle$	not affected	not affected	not affected
$\langle T_3\vert\big[W,T_4\big]\rangle$	not affected	not affected	not affected
$\langle L_3\vert\, e^{-T}He^{T}\rangle$	−0.023	−0.081	−0.021
$\langle L_4\vert\big[F,T_4\big]\rangle$	−0.598	−0.260	−0.032
$\langle L_4\big[W,T_3\big]\rangle$	0.202	0.540	0.801
$\frac{1}{2}\langle\Big[\big[W,T_2\big],T_2\Big]\rangle$	0.603	0.749	−0.792
approximations to L3: use of Eqs. (21)–(22)			
$\langle L_3\vert\, e^{-T}He^{T}\rangle$	−0.024	−0.082	−0.021
(other terms not affected)			

SI.13 Molecules used in benchmark calculations in Sec. 3.3

The list of molecules reads: borane (BH_3), acetylene (C_2H_2), methane (CH_4), carbon dioxide (CO_2), carbon monoxide (CO), formaldehyde (H_2CO), hydrogen peroxide (H_2O_2), water (H_2O), isocyanic acid (HNCO), hydrogen cyanide (HCN), formic acid (HCOOH), hydrogen fluoride (HF), nitrous oxide (N_2O), dinitrogen (N_2), ammonia (NH_3). Geometries of all molecules in Cartesian coordinates (`*.xyz` file format) are attached to this document in a

separate archive file (`geometries.tar`). The geometries are given in the units of ångströms.

SI.14 Comparison of computational timings

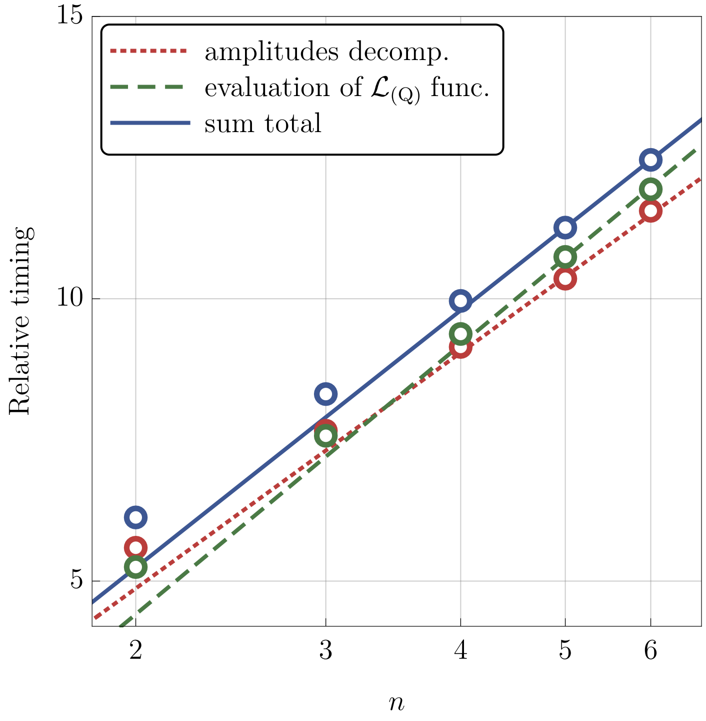

Figure 1: Relative timings of two major computational steps of the proposed formalism (cc-pVTZ basis set) for linear alkanes (C_nH_{2n+2}) as a function of the chain length, n. Logarithmic scale is used on both axes.

SI.15 Errors in absolute energies with standard auxiliary basis sets

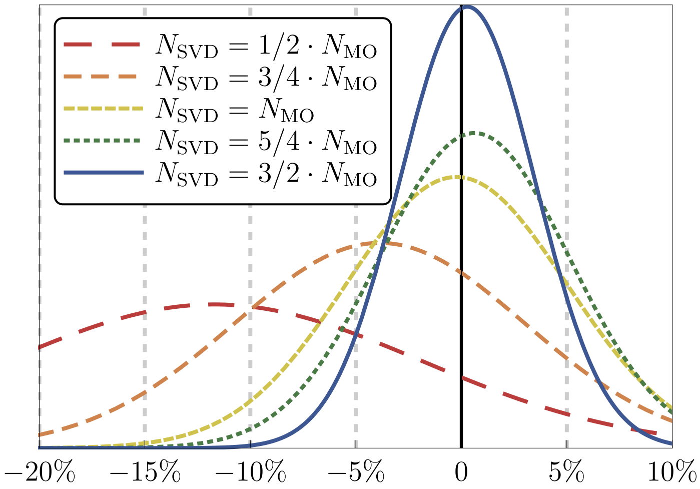

Figure 2: Distribution of relative errors (in percent) in the (Q) correction obtained using the $\mathcal{L}_{(\mathrm{Q})}$ functional (cc-pVTZ basis set) for several representative values of the N_{SVD} parameter, see the legend. For each value of N_{SVD}, the parameter N_{qua} is set to $\frac{2}{3}N_{\mathrm{SVD}}$. The exact CCSDT(Q) results are used as a reference. The symbol N_{MO} denotes the total number of orbitals in a given system. The data is the same as in Fig. 4 of the main text, but here the standard cc-pVTZ-RIFIT auxiliary basis set for density-fitting approximation is used.

SI.16 Optimized structures of bullvalene

Equilibrium structure (Å)

C	-0.00000026	-0.00000164	1.54686903
C	1.24512375	-0.71887260	1.07290296
C	-0.00000004	1.43774588	1.07290166
H	-0.00000024	-0.00000096	2.63473419
H	1.91483260	-1.10552930	1.83035942
H	-0.00000019	2.21105810	1.83035942
C	0.00000049	1.79357827	-0.21297427
C	0.00000020	0.88349125	-1.36415025
H	0.00000113	2.84940415	-0.45539106
C	1.55328396	-0.89678782	-0.21297326
C	0.76512701	-0.44174612	-1.36415096
C	-1.24512412	-0.71887347	1.07290329
C	-1.55328411	-0.89678831	-0.21297319
H	-1.91483284	-1.10553025	1.83035995
C	-0.76512696	-0.44174596	-1.36415066
H	-1.19719093	-0.69119721	-2.32223685
H	2.46765651	-1.42470002	-0.45538983
H	1.19719050	-0.69119757	-2.32223737
H	-2.46765647	-1.42470065	-0.45538991
H	0.00000000	1.38239496	-2.32223659

Transition state (Å)

C	-0.00000775	1.49877862	0.46663860
C	-0.00000223	0.66258578	1.70968107
C	-1.06670622	1.24182624	-0.55996813
H	-0.00001703	2.55046682	0.73781474
H	-0.00002181	1.19456621	2.65297194
H	-1.44371354	2.11428714	-1.07498143
C	-1.52080192	-0.00003760	-0.96847542
C	-1.06663128	-1.24188788	-0.55993548
H	-2.23988371	-0.00007025	-1.77895814
C	0.00001142	-0.66248017	1.70971121
C	0.00002120	-1.49876939	0.46673105
C	1.06669078	1.24184667	-0.55997272
C	1.52079958	-0.00000811	-0.96849235
H	1.44367609	2.11431504	-1.07498976
C	1.06665233	-1.24186553	-0.55995076
H	1.44338099	-2.11432676	-1.07516854
H	0.00000315	-1.19441480	2.65302850
H	0.00003715	-2.55043008	0.73802156
H	2.23986936	-0.00002614	-1.77898572
H	-1.44335657	-2.11435581	-1.07514451
